# Hardened Properties of Eco-Friendly Concrete Using Crushed Waste Shells as All Fine Aggregates

**DOI:** 10.3390/ma19214555

**Published:** 2026-10-28

**Authors:** Ruiwen Liu, Yingdi Liao, Bo Da, Junhao Fan, Da Chen, Xiaotong Yu

**Affiliations:** 1 Key Laboratory of Coastal Disaster and Defence of Ministry of Education, Hohai University, Nanjing 210098, China; 2 College of Mechanics and Engineering Science, Hohai University, Nanjing 210098, China; 3 The National Key Laboratory of Water Disaster Prevention, Nanjing Hydraulic Research Institute, Nanjing 210029, China; 4 Yangtze Institute for Conservation and Development, Hohai University, Nanjing 210098, China; 5 Centre for Offshore Foundations Systems, School of Earth and Oceans, University of Western Australia, Perth 6009, Australia

**Keywords:** crushed waste shells, eco-friendly concrete, pre-absorption, properties

## Abstract

**Highlights:**

- Crushed waste shells (CWS) can fully replace river sand in eco-friendly concrete with different strength grades and satisfactory workability.
- The mechanical and durability properties of CWS concrete were investigated and compared with those of ordinary river sand concrete.
- The internal curing mechanism of CWS aggregates for enhancing concrete performance was clarified.

**Abstract:**

Considering the environmental problems arising from random disposal of waste shells and the overexploitation of river sand, crushed waste shells (CWS) can be recycled and used as fine aggregates in eco-friendly concrete production. Nevertheless, existing studies on CWS concrete still present notable limitations, including a low optimal replacement level and poor workability caused by the high water absorption of CWS. This study primarily explored the feasibility of 100% CWS replacement of fine aggregates for producing eco-friendly concrete with different strength grades and satisfactory workability. With pre-absorption treatment applied to CWS aggregates, three groups of eco-friendly CWS concrete (C30, C40 and C50) were made instead of direct sand replacement based on ordinary river sand concrete. The hardened properties of CWS concrete, including mechanical strength, durability-related transport characteristics, pore structure, mineral composition and microstructure, were systematically investigated and compared with those of ordinary river sand concrete. This study provides a technical basis for the efficient recycling of waste shell resources and the practical engineering application of eco-friendly CWS concrete.

## 1. Introduction

While concrete underpins global infrastructure development, the concrete sector is confronted with twofold challenges: the exhaustion of natural aggregate resources and increasing ecological and environmental stresses [1]. River sand is one of the most widely used fine aggregate for conventional concrete, with an annual consumption exceeding 10.5 billion tons [2]. However, overexploitation of river sand has caused the mining rate to exceed the natural replenishment rate, leading to the depletion of natural river sand resources and exerting adverse impacts on aquatic habitats, riverbed morphology and groundwater levels [3]. Many national and regional governments (e.g., China and India) have recognized the numerous adverse effects of excessive river sand extraction on the environment and have issued policies to impose tight restrictions on river sand extraction [4]. Against this backdrop, researchers have been driven to explore alternative fine aggregates to replace river sand in concrete. Among various technical routes, reutilization of solid wastes serves as a key pathway for achieving sustainability in concrete production and has become a major research focus within eco-friendly concrete research [5,6].

In the meantime, seashells belong to marine resources with fast accumulation rates, especially in coastal countries like China, Japan, South Korea, Thailand, and other areas. Owing to the rapid expansion of shellfish aquaculture, the shellfish output of China reached 15 million tons in 2020 [7]. As stated in literature, every kilogram of oysters or scallops for consumption yields roughly 700 g of shell waste [8]. However, these waste shells have little or no commercial value, with merely 30% of the total material being recycled for secondary utilization [9]. Most discarded shells are randomly buried in farmland or open landfills, releasing offensive odors, attracting pests, and eventually causing severe ecological environment and public health problems [10]. Fortunately, former researchers had already noticed that calcium carbonate serves as the primary constituent of waste shells, accounting for almost 96% of total weight [11]. This stable chemical characteristic is similar to that of traditional aggregates, which are not involved in hydration reactions inside cement-based materials. As a result, crushed waste shells (CWS) have been proposed as an alternative aggregate for concrete fabrication to address the above-mentioned issues stemming from excessive river sand extraction and discarded shell accumulation.

Numerous investigations have been carried out to date to explore the potential of utilizing CWS as aggregates for concrete manufacture [12,13,14]. Yoon et al. [9] observed that CWS can act as a substitute to make up for the shortage of natural aggregate in cement-based materials. Subsequently, numerous researchers have concentrated on the effects of particle size and CWS aggregate proportion on the properties of concrete mixtures. Existing studies present inconsistent conclusions on the effect of particle size. Martinez-Garcia et al. [15] observed that using seashells as fine aggregate replacements led to a more obvious decline in compressive strength than their use as coarse aggregate replacements. In contrast, Yoon et al. [9] and Eo and Yi [16] found that concrete containing finer CWS aggregates exhibited a smaller drop in compressive strength compared with those using coarser CWS aggregates. Research conducted by others [17] further confirmed this trend, stating that concrete incorporating fine CWS aggregates generally had higher compressive strength than those containing coarse particles, due to reduced void content and enhanced aggregate-paste interfacial bonding. In view of its morphology, CWS is better suited for replacing fine aggregates instead of coarse aggregates in concrete production.

Nevertheless, several problems still need to be resolved in present research, which restricts the utilization of CWS as fine aggregate for river sand replacement in concrete. Most previous studies used CWS to replace fine aggregates following the mix proportion of conventional river sand concrete [18,19]. Accordingly, researchers identified that an optimal level of CWS aggregate replacement existed with regard to strength performance. In previous investigations, the mass replacement ratio of CWS fine aggregate for river sand was mainly between 5% and 60% [17]. Studies [20,21] have shown that concrete incorporating 5–20% CWS typically exhibits superior compressive strength compared with conventional concrete, while a further rise in replacement ratio causes an obvious strength reduction [22]. Only a small number of studies have attempted full 100% replacement. The concrete produced in these cases exhibited inferior mechanical properties and could not satisfy engineering criteria. According to literature [15], the compressive strength dropped by up to 72% when CWS completely replaced river sand as fine aggregate. Apart from the restriction related to the optimal replacement level, the physical properties of CWS also bring another challenge. Numerous studies have proven that the incorporation of CWS as fine aggregate in concrete lowers workability, and the workability of fresh concrete generally declines with rising CWS replacement level [22]. This is mainly attributed to the irregular morphology and high specific surface area of CWS particles, which raise water absorption and thus exert negative impacts on the workability of fresh mixtures.

**Figure 1:**
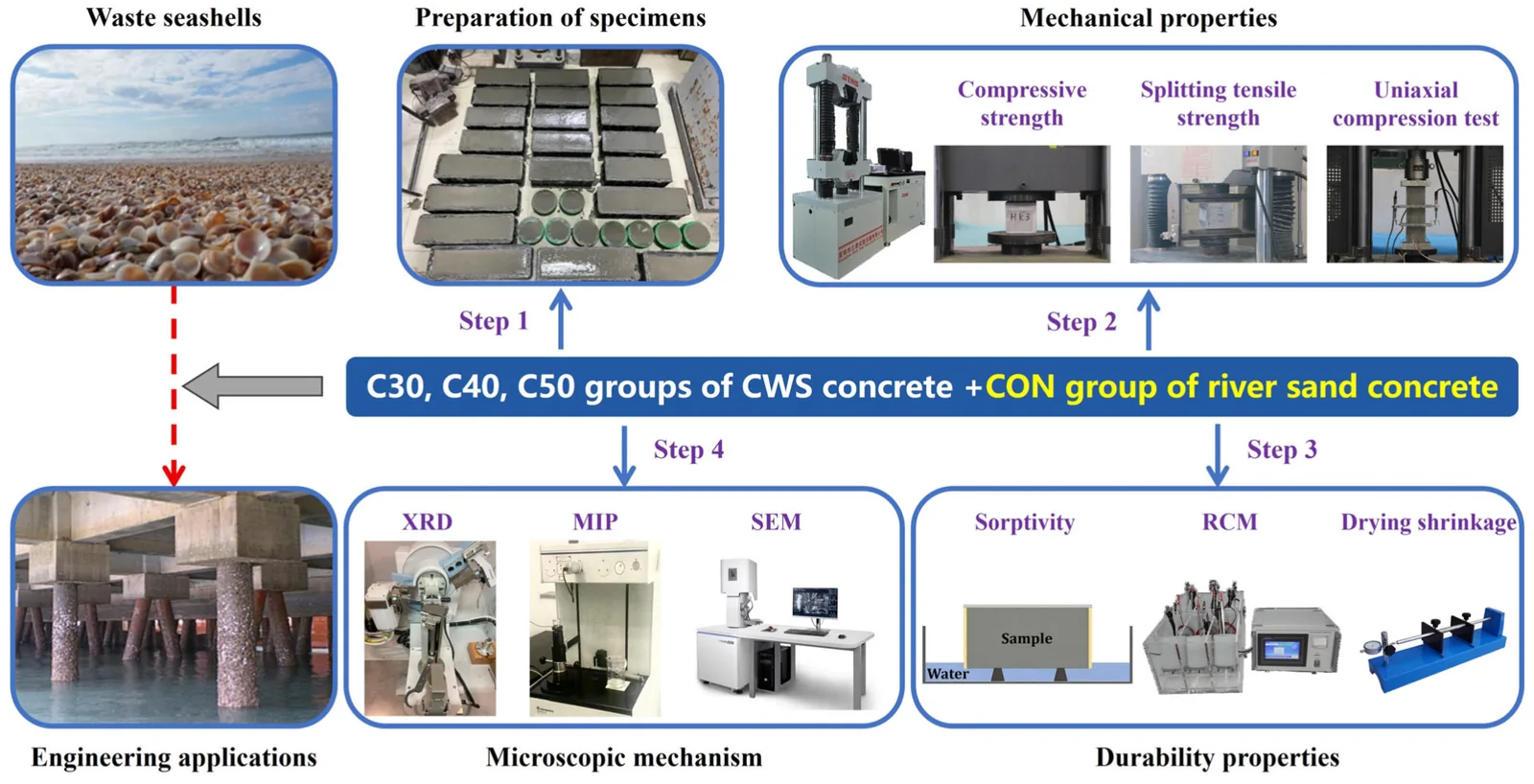
Framework of the present study.

From the foregoing literature review, it is clear that existing investigations on CWS concrete exhibit multiple limitations, including a relatively low optimal replacement ratio and poor workability resulting from the high water absorption of CWS. Hence, this research was carried out to explore the feasibility of fully utilizing CWS as all fine aggregate in the preparation of eco-friendly concrete with different strength grades. Three groups of CWS concrete (C30, C40 and C50) were made considering the pre-absorption method of CWS fine aggregate in this study. All the CWS concrete mixtures adopted the optimized mix proportions and sand ratios from prior research, rather than directly replacing river sand from conventional concrete. The hardened properties of CWS concrete, including mechanical strength, durability-associated transport characteristics, pore size distribution, mineral phases and microstructural features, were investigated and compared with those of the control concrete using natural river sand as the full fine aggregate. The overall research procedure adopted in this work is illustrated in Figure 1. This investigation offered a thorough comprehension of the hardened performance of eco-friendly concrete containing CWS as all fine aggregates, which will facilitate the effective recycling of waste shell resources and the practical engineering implementation of eco-friendly CWS concrete.

## 2. Raw Materials

### 2.1. Cement

In this work, Ordinary Portland cement (OPC) of strength grade P.O. 42.5 was adopted as the binding material, which complies with the Standard GB 175-2023 [23]. The primary oxide constituents of the cement are presented in Table 1.

### 2.2. Fine Aggregates

**Figure 2:**
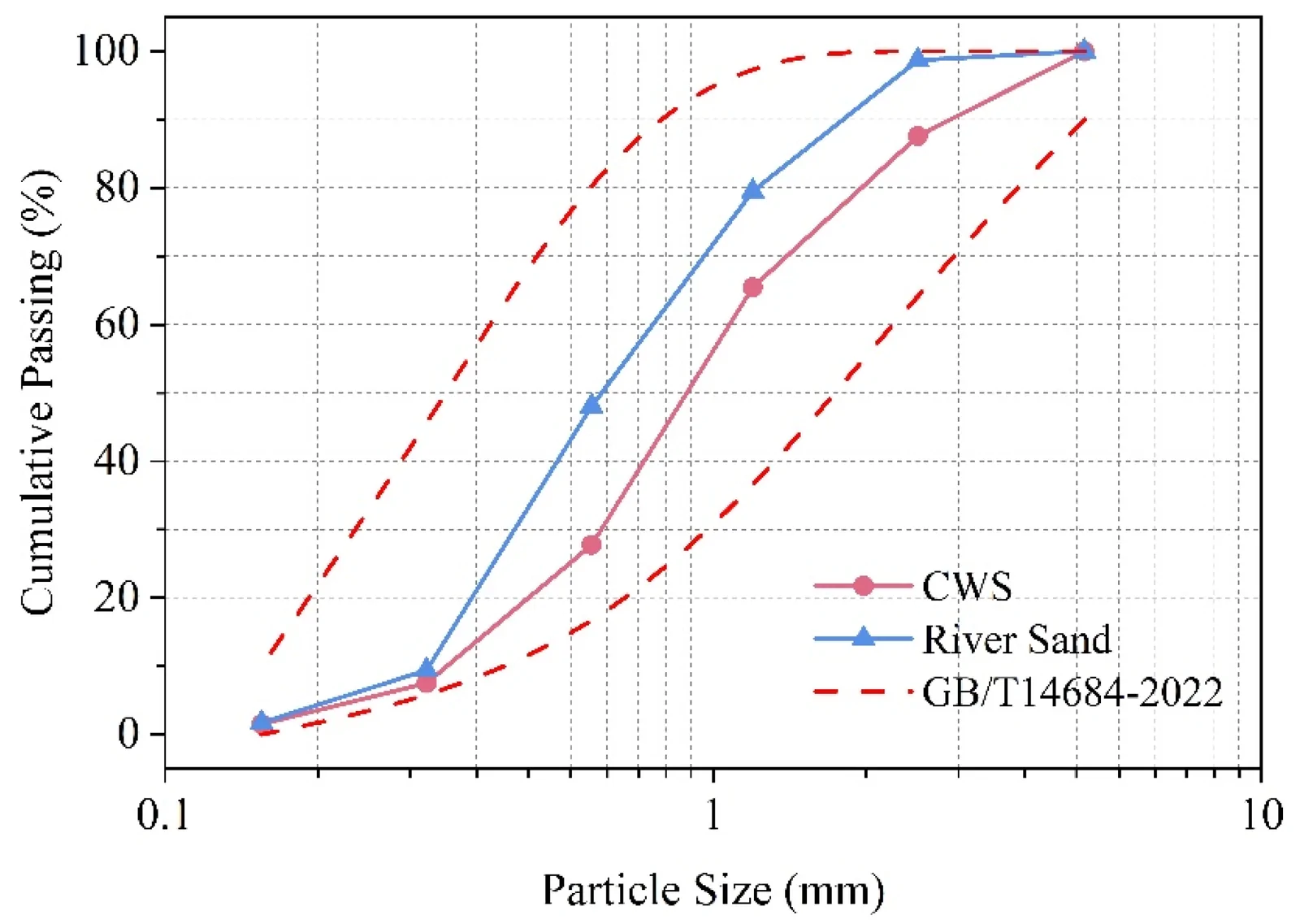
Particle size distributions of fine aggregates.

Natural river sand and crushed waste shells (CWS) were adopted as fine aggregates in this work, both of which were obtained from the eastern coastal regions of China and possessed a maximum particle size of 5 mm. Waste shells were first washed to remove surface residues including soluble salts, and then crushed to produce CWS aggregates. River sand was adopted as the 100% fine aggregate for ordinary concrete mixtures in the control group, whereas CWS served as the 100% fine aggregate for all CWS concrete mixtures. The particle size distribution profiles of river sand and CWS fine aggregates are shown in Figure 2. The fineness moduli of CWS and river sand are 3.0 and 2.7, respectively. According to Standard GB/T 14684-2022 [24], these two types of fine aggregates are classified as medium sand. The specific gravity of river sand and CWS fine aggregate is 2.58 and 2.39, respectively, and their bulk density is 1507 kg/m^3^ and 1464 kg/m^3^, respectively.

### 2.3. Coarse Aggregates

**Figure 3:**
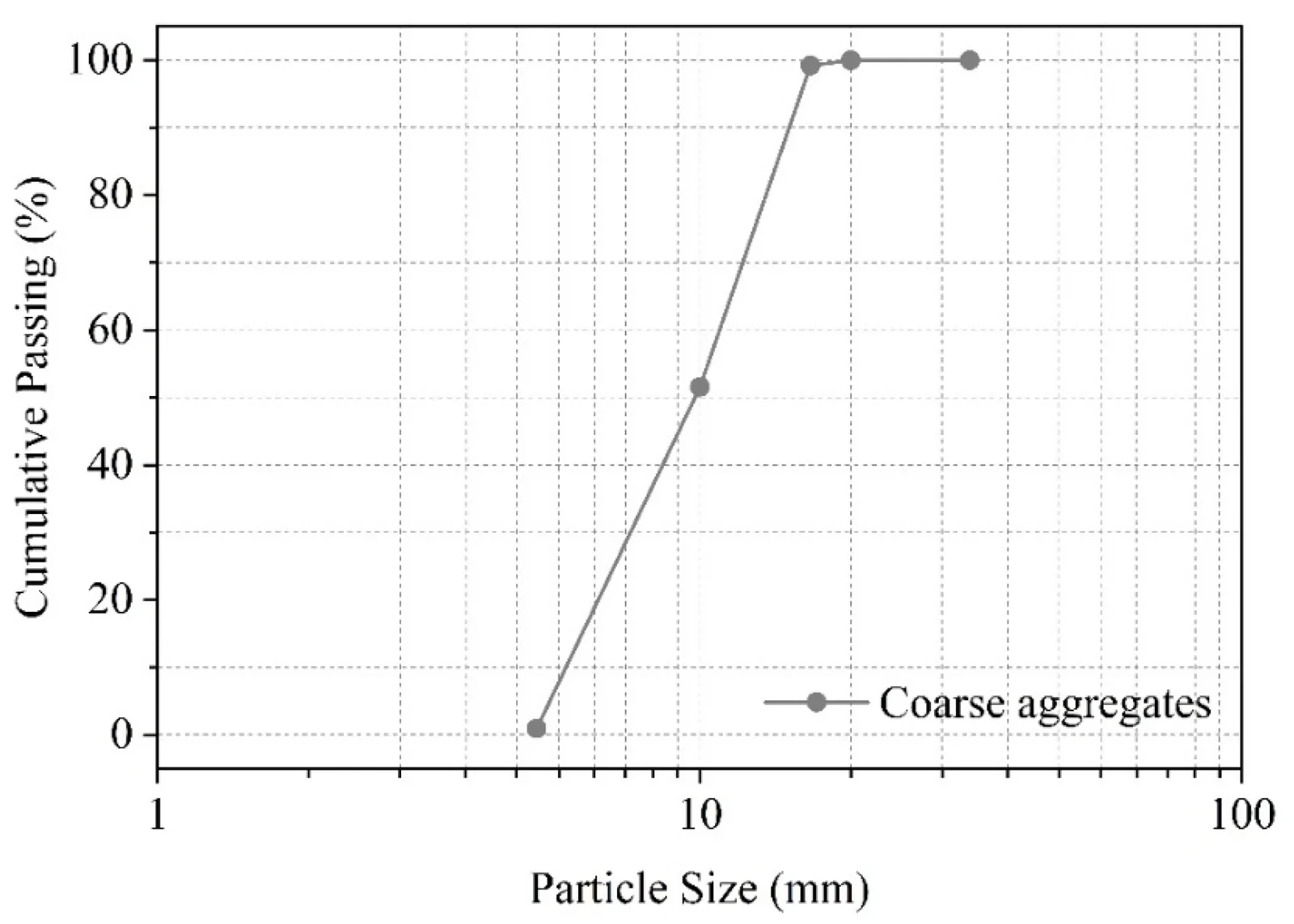
Particle size distributions of coarse aggregates.

The coarse aggregate used for all concrete mixtures was limestone of sedimentary origin, with a maximum particle size of 20 mm. The natural gravel was subjected to sieving to remove impurities such as leaves mixed within aggregates before the experiment. The particle size distribution of coarse aggregates is illustrated in Figure 3. The specific gravity of coarse aggregate is 2.68, and its bulk density is 1545 kg/m^3^.

### 2.4. Superplasticizer and Mix Water

A sulfonated naphthalene formaldehyde (SNF) superplasticizer supplied by Shanghai Chenqi Chemical Co., Ltd. (Shanghai, China) was used, which features a solid content higher than 96%, with sodium sulfate and chloride ion contents less than 20% and 0.4%, respectively. Laboratory tap water was employed for concrete mixing.

## 3. Experiment Programs

### 3.1. Mixture Proportions

Mix proportions of all concrete specimens are summarized in Table 2. CWS serves as a typical porous marine aggregate, which features abundant internal pores (including both connected and closed pores), a large specific surface area, and high water absorption. The detailed mix-proportion parameters of CWS concrete were selected with reference to Standard JGJ/T 12-2019 [25], combined with existing research on concrete fabricated with porous marine aggregates. Final mixture proportions were determined by trial mixing considering the material properties of CWS.

Three groups of CWS concrete designed for different strength grades were denoted as C30, C40 and C50, respectively. Comparable apparent densities (approximately 2400 kg/m^3^) were maintained among these three CWS mixtures. CWS was adopted as the 100% replacement for fine aggregate in all CWS concrete mixtures, and the sand ratio was set to 0.45–0.5 to maximize the utilization of waste shell resources. Following the existing preparation method for coral-sand concrete, pre-absorption treatment was performed on CWS fine aggregate at a fixed water-absorption rate of 4% to improve the workability of CWS concrete. The adopted CWS aggregate has a saturated water absorption of approximately 8%. Based on our prior investigations, this moderate pre-absorption level of 4% was selected. As shown in Table 2, there are both total and net water-cement ratios (w/c), where the net w/c excludes the water used in pre-absorption treatment. From the perspective of the excess-paste theory, porous marine aggregates or lightweight aggregates such as coral-sand, which possess abundant internal pores and weak interfacial bonding, inherently require higher cementitious dosage to form adequate paste wrapping and matrix compaction [26,27]. Increasing cement content has been widely recognized as a feasible laboratory design strategy to compensate for the intrinsic defects of porous aggregates and achieve designated strength grades [28,29]. Accordingly, under the given constraints of apparent density and sand ratio range, the cement dosage was progressively raised, while the total water-cement ratio was correspondingly lowered to meet the incremental strength requirements from C30 to C50. This study aims to verify the feasibility of producing concrete with different strength grades using 100% CWS as fine aggregate and evaluate its performance. It is not intended to explore aggregate effects at a fixed water-cement ratio, nor to investigate the impacts of mixture parameters on concrete performance.

Meanwhile, a control group, denoted as CON, was prepared with natural river sand as all fine aggregates, adopting the same mixing proportion as the C30 group of CWS concrete. This control group was intended to evaluate the influences of 100% CWS replacement for natural river sand fine aggregate on concrete performance. Since the C40 and C50 CWS-concrete series featured elevated cement contents that modified the cementitious system, these groups were not compared against the CON group. All CWS-concrete groups (C30, C40, and C50) employed the same kind of aggregate and exhibited comparable apparent densities, enabling comparative analysis of performance evolution for CWS concrete across different target strength grades. It should be emphasized that the observed performance variations arise from the coupled effects of the CWS aggregate and increased cement dosage and cannot be ascribed to any single-factor contribution. This study did not aim to investigate the effect of aggregate type using the C40 and C50 series, so the corresponding river-sand concrete groups were not prepared for these two strength grades.

Moreover, conventional SNF superplasticizer was adopted as the chemical admixture in all mixtures. The slump values for each concrete group are also listed in Table 2.

### 3.2. Preparation of Concrete Specimens

The mixing procedures were identical for all specimens following the Chinese Standard SL/T 352-2020 [30]. The CWS fine aggregate was first subjected to pre-absorption. The pre-absorption water quantity was calculated from the mass of CWS aggregate and the designated pre-absorption rate, which was fixed at 4% of the CWS fine aggregate mass. During the pre-absorption period, powdered chemical admixture was completely dissolved in mixing water. Cement and all coarse aggregates were first premixed for 120 s. Subsequently, water containing dissolved admixture was added, followed by continuous stirring for another 90 s. A 30 s mixing interval was then arranged. After an additional 60 s of mixing, workability tests were promptly performed on fresh concrete before it was cast into metallic and plastic molds in two layers. Specimens were compacted using a vibration table to expel entrapped air. After casting, plastic film was used to cover samples and reduce water evaporation. After 24 h indoor curing, all specimens were demolded and then placed in a standard water curing tank maintained at 23 ± 1 °C until the test ages.

### 3.3. Testing Methods

#### 3.3.1. Compressive Strength Test

The compressive strength of concrete specimens was determined in compliance with Chinese Standard SL/T 352-2020 [30]. Average compressive strength values were acquired from triplicate samples for each group at curing ages of 3, 7, 28 and 90 d. The compressive strength were carried out on 100 mm × 100 mm × 100 mm specimens using an electro-hydraulic servo universal testing machine, as illustrated in Figure 1. The compressive strength of each specimen can be calculated according to Equation (1).

(1) $$f_{c}=\frac{F}{A}$$

where *f*_c_ denotes the compressive strength of the specimen, MPa; *F* represents the failure load of the specimen, N; *A* refers to the bearing area of the specimen, mm^2^.

#### 3.3.2. Splitting Tensile Strength Test

At curing ages of 3, 7, 28 and 90 d, splitting tensile strength tests were carried out on three cube specimens with a side length of 100 mm, and the corresponding values were derived from the average of test results. An electro-hydraulic servo universal testing machine was employed for splitting tensile strength measurements at a loading rate of 50 N/s. The specimen arrangement prior to loading is presented in Figure 1. In the test process, soft gaskets were attached to both sides of the specimen to mitigate the influence of stress concentration. According to Chinese Standard SL/T 352-2020 [30], the splitting tensile strength of the specimen can be determined via Equation (2).

(2) $$f_{st}=\frac{2F}{\pi A}=0.637\frac{F}{A}$$

where *f*_st_ denotes the splitting tensile strength of the specimen, MPa; *F* represents the failure load of the specimen, N; *A* refers to the splitting surface area of the specimen, mm^2^.

#### 3.3.3. Uniaxial Compression Test

**Figure 4:**
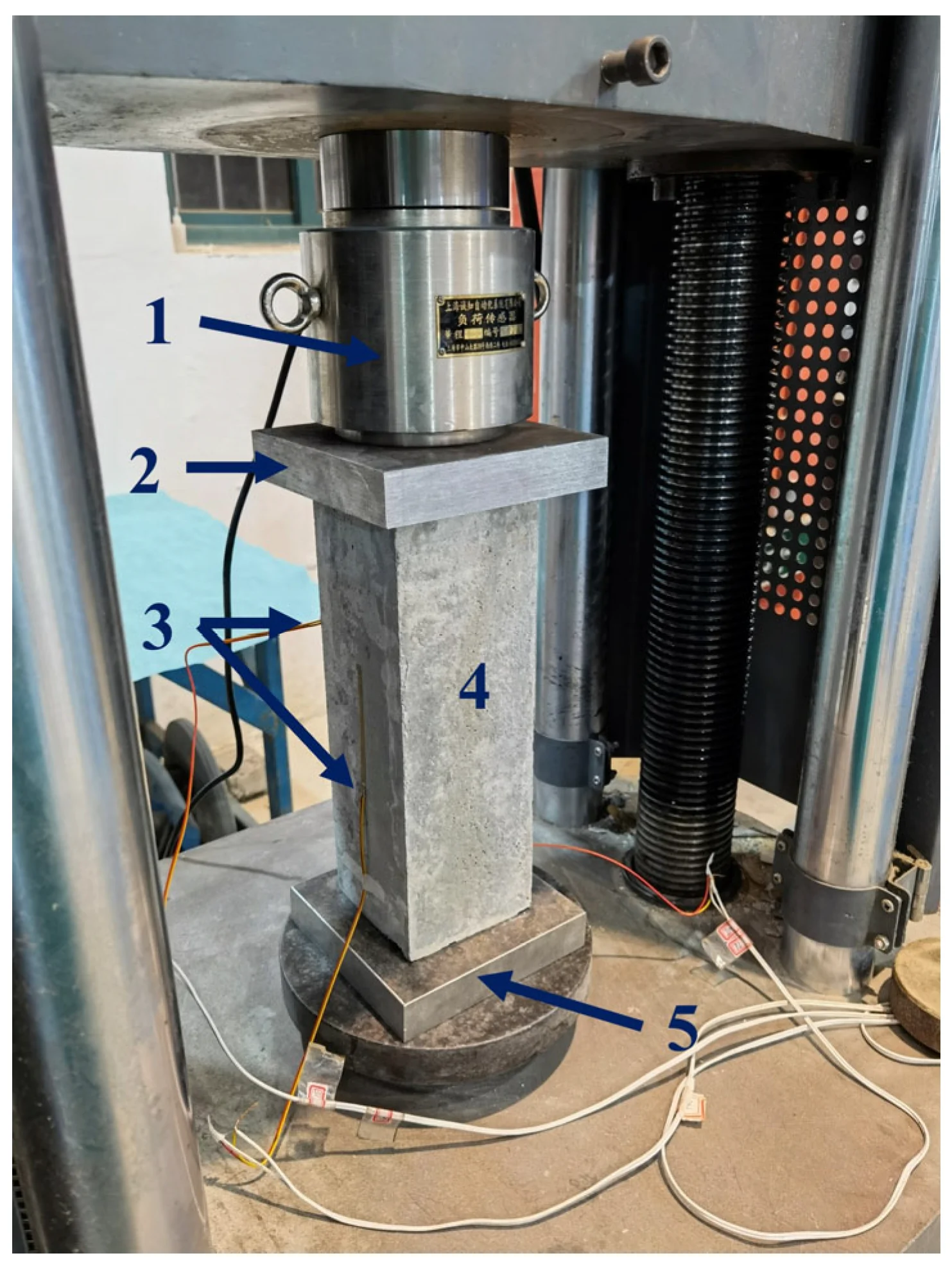
Uniaxial compression test. (1: Load sensor; 2: Upper pad; 3: Strain gauge; 4: Specimen; 5: Lower pad.).

The axial compressive strength, static elastic modulus and Poisson’s ratio for each concrete group were measured via uniaxial compression tests following the Chinese Standard GB/T 50081-2019 [31]. At curing ages of 28 d and 90 d, prismatic specimens sized 100 mm × 100 mm × 300 mm were used for measurements, and the final results were averaged from three replicate specimens. As shown in Figure 4, to ensure experimental accuracy, 120-80AA foil resistance strain gauges were pasted parallel to the axial direction on both sides of each specimen, and 120-50AA foil resistance strain gauges were arranged along the transverse direction. They were separately used to monitor the longitudinal and transverse deformation of specimens, respectively. Meanwhile, the load was measured by a calibrated 200 t force sensor. Cyclic loading procedures including centering and preloading were carried out, and the adopted loading system was the same as compressive strength test. During the whole test process, the DH3820 dynamic data acquisition system was linked to a computer to record real-time stress and strain data.

Finally, the axial compressive strength of the specimen can be calculated according to Equation (3).

(3) $$f_{cp}=\frac{F}{A}$$

where *f*_cp_ is defined as the axial compressive strength of the specimen, MPa; *F* is the failure load of the specimen, N; *A* stands for the stress-bearing area of the specimen, mm^2^.

The static elastic modulus of the specimen can be calculated according to Equation (4).

(4) $$\left\{ \begin{array}{c} E_{c}=\frac{F_{a}-F_{0}}{A}\times \frac{L}{\Delta n}\\ \Delta n=\varepsilon_{a}-\varepsilon_{0} \end{array}\right.$$

where *E*_c_ denotes the static elastic modulus of the specimen, MPa; *F*_a_ represents the control load, which the stress is 1/3 *f*_cp_, N; *F*_0_ refers to the initial load, which the stress is 0.5 MPa, N; *A* is the bearing area of the specimen, mm^2^; *L* stands for the strain gauge length, mm; ∆*n* indicates the deformation variation of the specimen from *F*_0_ to *F*_a_, mm; $\varepsilon_{a}$ and $\varepsilon_{0}$ denote the average bilateral deformations of specimens under *F*_a_ and *F*_0_, mm.

Poisson’s ratio of the specimen can be calculated according to Equation (5).

(5) $$\mu =\frac{\varepsilon_{\mathrm{tr}}}{\varepsilon_{p}}$$

where $\varepsilon_{tr}$ represents the transverse strain of the specimen; $\varepsilon_{p}$ represents the longitudinal strain of the specimen.

#### 3.3.4. Sorptivity Test

Specimens with a dimension of Φ 100 × 50 mm were prepared for the sorptivity test at curing ages of 28 d and 90 d. The entire test procedure was implemented in strict accordance with the Standard ASTM C1585-20 [32]. Prior to testing, the oven-dried specimens were coated with waterproof epoxy on their lateral surfaces, while the top surfaces were covered and sealed with plastic film. The initial weight of each specimen and the diameter of the water-absorbing surface were accurately recorded before the commencement of the test. Subsequently, all treated samples were placed on supporting brackets inside a water tank, with the water level maintained 3–5 mm above the bracket surface. The variation in specimen mass was measured at predetermined time intervals (*t*), including 60 s, 5 min, 10 min, 20 min, 30 min, 1 h, 3 h, 4 h, 5 h, 6 h, 12 h, 1 d, 2 d, 3 d, 4 d, 5 d, 6 d, and 7 d. The sorptivity of the concrete sample can be obtained as expressed:

(6) $$I=\frac{m_{t}}{a\times d}$$

where *I* is the absorption, mm; *m_t_* is the change in specimen mass at the time *t*, g; *t* represents the absorption duration, s; *a* is the exposed area of the specimen, mm^2^; *d* is the density of the water, g/mm^3^.

#### 3.3.5. Rapid Chloride Migration Test

According to Chinese Standard GB/T 50082-2024 [33], rapid chloride migration (RCM) tests were performed to assess chloride penetration resistance. The measurement was conducted using cylindrical specimens (Φ100 mm × 50 mm) at 28 d and 90 d using an RCM-NTB chloride ion diffusion analyser (Figure 1). Before the test, the thickness of each specimen was measured and recorded with vernier callipers. After testing, specimens were fractured using a compression tester. The fractured surfaces were evenly sprayed with 0.1 mol/L AgNO_3_ solution and maintained for 15 min to enable colour development. Ultimately, the chloride ion migration coefficient can be calculated using Equation (7).

(7) $$D_{RCM}\;=\;\frac{0.0239\times (273+T)}{(U-2)t}\;\left(\;x_{d}-0.0238\sqrt{\frac{(273+T)L\cdot x_{d}}{U-2}}\;\right)$$

where *D*_RCM_ denotes the chloride ion migration coefficient, m^2^/s; *T* represents the average temperature in the anode from the start to the end of the test, K; *U* refers to the applied test voltage, V; *L* stands for the specimen thickness, m; *x_d_* indicates the average chloride penetration depth, m; *t* is the test duration, s.

#### 3.3.6. Drying Shrinkage Test

Drying shrinkage measurements were conducted on prismatic specimens sized 100 mm × 100 mm × 515 mm, complying with Chinese Standard GB/T 50082-2024 [33]. Once the concrete specimens were demolded, their original lengths were immediately measured and recorded. All samples were then stored in a constant environmental condition and taken out for measurement until specified testing ages at 14 d, 28 d, 45 d, 90 d, 120 d, 150 d, 180 d, 210 d, and 240 d. A dimensionless parameter $\varepsilon_{t}$ was defined to assess the percentage drying shrinkage, as given below.

(8) $$\varepsilon_{t}=\frac{L_{0}-L}{L_{0}}\times 100\%$$

where $\varepsilon_{t}$ denotes the specimen length change at a given age, %; *L*_0_ represents the initial length of the specimen, mm; *L* refers to the specimen length at each measurement, mm.

#### 3.3.7. Mercury Intrusion Porosimetry (MIP) Test

Mercury intrusion porosimetry (MIP) is a prevalent technique for characterizing the pore size distribution of cementitious materials. The basic principle is to measure the amount of mercury entering the pores under different external pressures to obtain the pore volume of the corresponding pore size. Before the test, specimens were dried thoroughly to expel free water within internal pores. Small fragments weighing 1–2 g were prepared for the MIP test. All tests were conducted using a Micromeritics Autopore IV 9510 porosimeter. The surface tension and contact angle of mercury were taken as 0.485 N/m and 130°, respectively. The applied pressure was regulated in the range of 0.2–50000 psi, covering detectable pore diameters ranging from 3 nm to 300 μm.

#### 3.3.8. Mineralogy and Microstructure Test

X-ray diffraction (XRD) testing can quickly determine the mineral composition of a substance. Since each type of substance has its own specific diffraction pattern, the intensity of the diffraction peak is related to the substance content of the sample to be tested. Based on the above principle, the sample can be qualitatively and semi-quantitatively analyzed by the XRD test. The XPert3 series X-ray diffractometer produced by Malvern Panalytical was used in combination with HighScore Plus 3.0e (3.0.5) software to perform phase analysis. Here the dried powder samples (particle size less than 75 μm) were used. The instrument parameters were set as follows: working voltage 40 kV, working current 40 mV, scanning angle 2-theta ranged from 0 to 90°, and scanning speed was determined according to the specific sample type.

A MIRA^3^ scanning electron microscopy (SEM) produced by Czech TESCAN Company was used to study the microstructure of the samples, equipped with Oxford EDS energy spectrum, which can perform element analysis. SEM observations were performed on freshly fractured surfaces of specimens using the secondary electron (SE) mode. To obtain high-quality images, all specimens were first coated with a thin carbon layer via the British Quorum Q150R Plus coater, and then mounted onto standard stubs with conductive tape to achieve adequate electrical conductivity.

## 4. Results and Discussion

### 4.1. Mechanical Properties

#### 4.1.1. Compressive Strength

**Figure 5:**
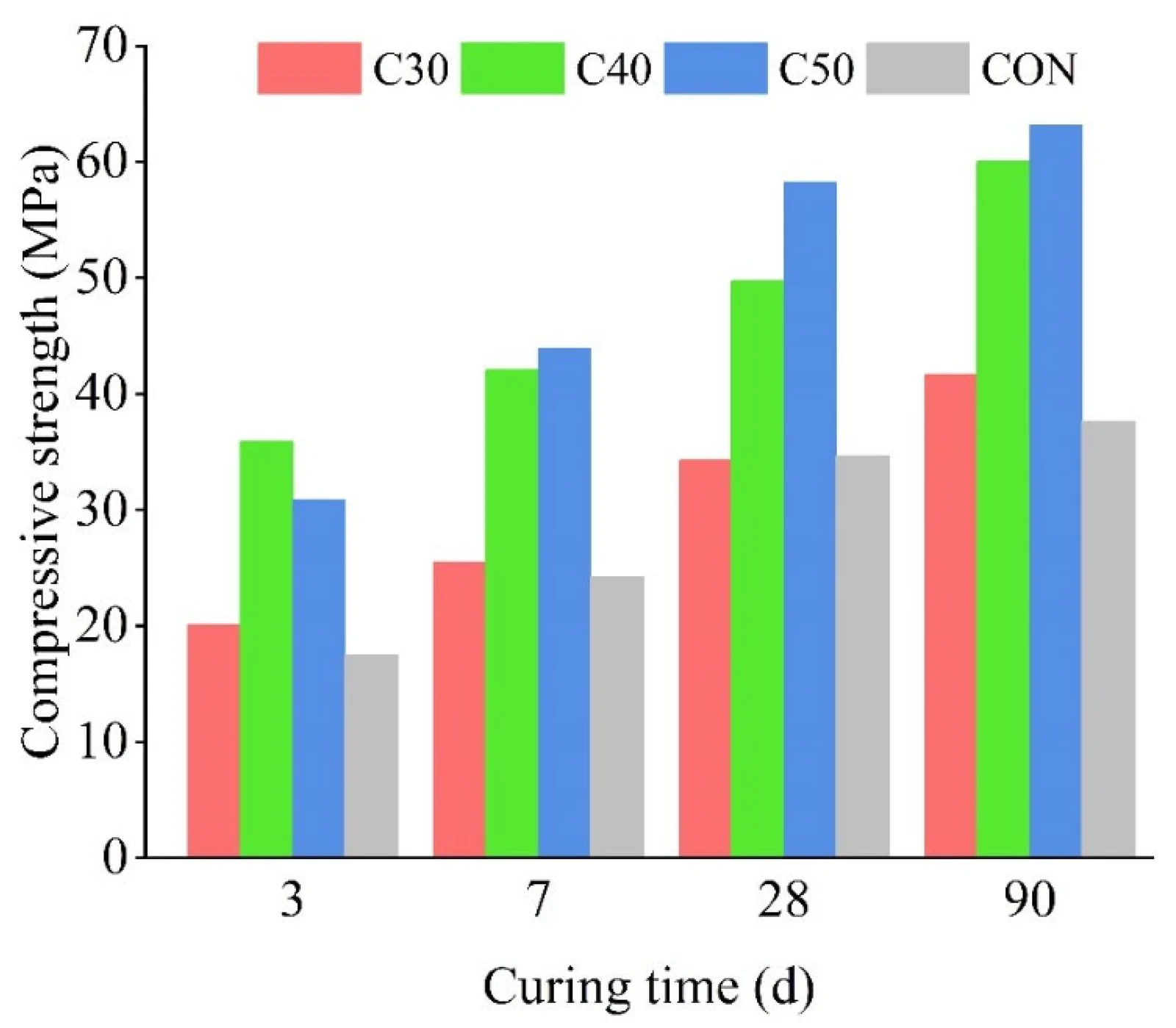
Development of compressive strength of concrete with curing age.

Figure 5 presents the evolution of compressive strength over time for all concrete mixtures. The development trend of compressive strength with curing age for CWS concrete was consistent with that of conventional river sand concrete. The compressive strength of all concrete specimens increased with the extension of the curing period. This phenomenon was primarily attributed to the continuous progress of the cement hydration. As hydration proceeded, the generation of hydration products increased, thereby refining the internal pore structure and improving the compactness of concrete matrices [34]. In addition, CWS concrete with different strength grades exhibited a compressive strength growth rate comparable to that of conventional river sand concrete. From 3 d to 28 d, the compressive strength of C30, C40, C50 and CON mixtures increased by 14.14 MPa, 13.85 MPa, 27.38 MPa and 17.15 MPa, respectively. In the subsequent period of 28 d to 90 d, the corresponding strength increases were 7.44 MPa, 10.32 MPa, 4.93 MPa and 2.94 MPa. For all concrete groups, the compressive strength rose rapidly at the early curing stage, followed by a gradual reduction in growth rate at later stages. This variation demonstrated that CWS concrete underwent vigorous hydration reactions in the early curing phase, and the hydration rate gradually declined over time, which was similar to the hydration characteristics of conventional river sand concrete.

It was also observed that the compressive strength of C30 group was marginally different from that of the control group at 28 d, where the compressive strength of C30 group was only 1.2% lower than the control group. Benefiting from the internal curing effect of CWS aggregates, the C30 group exhibited a compressive strength 10.9% higher than the CON group at 90 d. Meanwhile, given the identical mix proportions, CWS concrete and conventional river sand concrete had the same cementitious material content and water-cement ratio. However, CWS aggregates featured a rougher surface than conventional river sand. This led to tighter interfacial bonding between aggregates, cement paste and hydration products, so that CWS concrete achieved higher compressive strength under identical mix proportions [35].

#### 4.1.2. Splitting Tensile Strength

**Figure 6:**
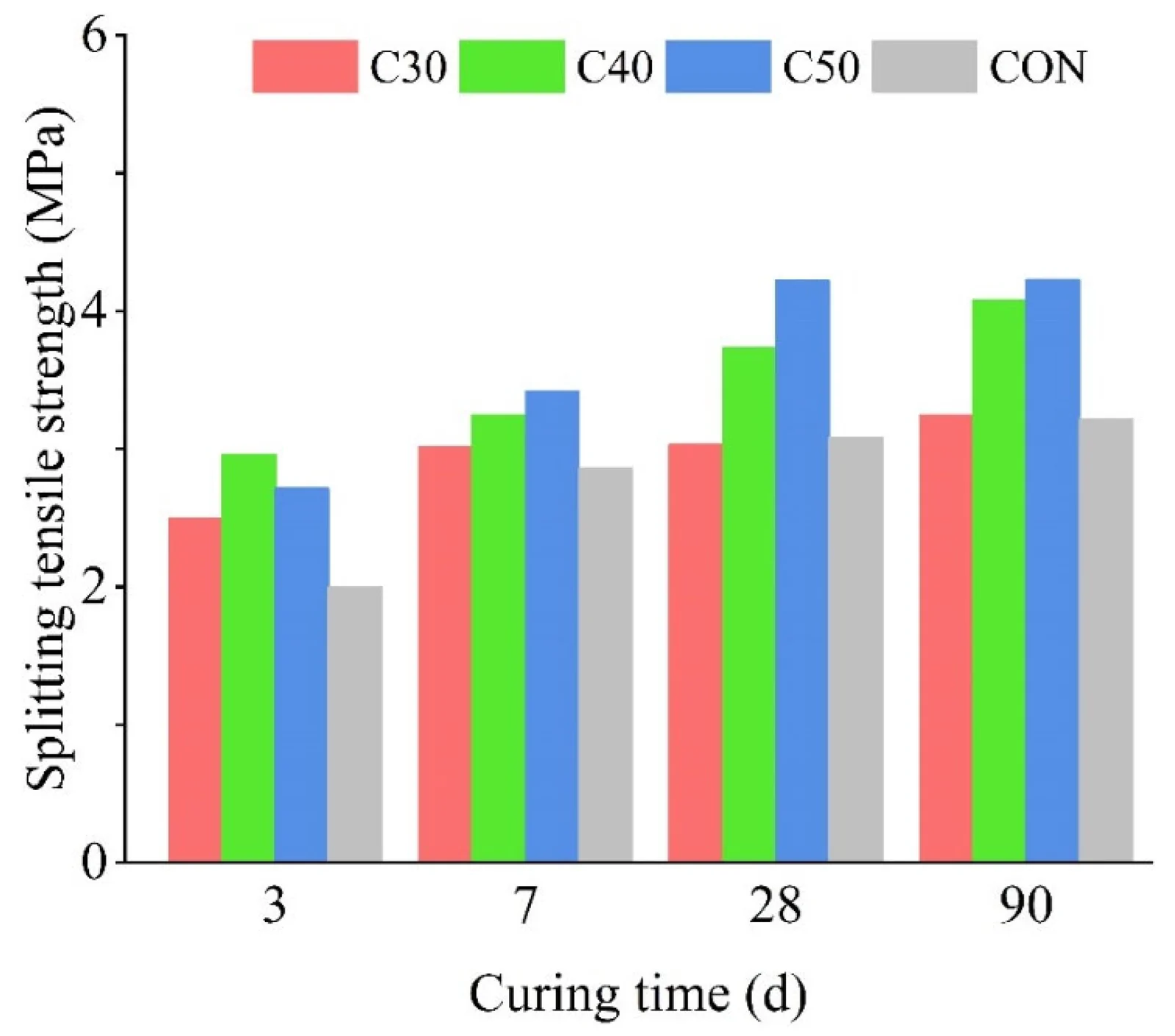
Development of splitting tensile strength of concrete with curing age.

Figure 6 presents the development of splitting tensile strength over time for all concrete mixtures. Compared with Figure 5, the evolution of splitting tensile strength over curing time followed a similar trend to that of compressive strength. Furthermore, CWS concrete exhibited a similar feature to conventional river sand concrete, i.e., a considerably lower splitting tensile strength relative to compressive strength. The results indicated that splitting tensile failure of both concrete mixtures originated primarily from interfacial cracking; thus, interfacial bonding strength dominated their tensile behaviour [36]. By contrast, compressive loads were mainly sustained by the cement paste matrix and aggregates, whereas the interfacial transition zone (ITZ) contributed secondarily and facilitated the full development of ultimate compressive capacity.

For mixtures prepared with identical mix proportions, CWS concrete exhibited distinctly higher splitting tensile strength than conventional river sand concrete at the early hydration stage, whereas the discrepancy became negligible at later ages. This trend differed slightly from the variation of compressive strength described above. At 3 d and 7 d, the C30 group of CWS concrete attained higher splitting tensile strength than the CON group of river sand concrete. At 28 d, the splitting tensile strength of the C30 and CON groups reached 3.03 MPa and 3.09 MPa, respectively. At 90 d, hydration proceeded continuously and further increased matrix density, while the inherent limit of interfacial bonding could not be changed. As a result, the splitting tensile strength increased slightly, with values of 3.24 MPa for the C30 group and 3.22 MPa for the CON group.

#### 4.1.3. Uniaxial Compression Results

**Figure 7:**
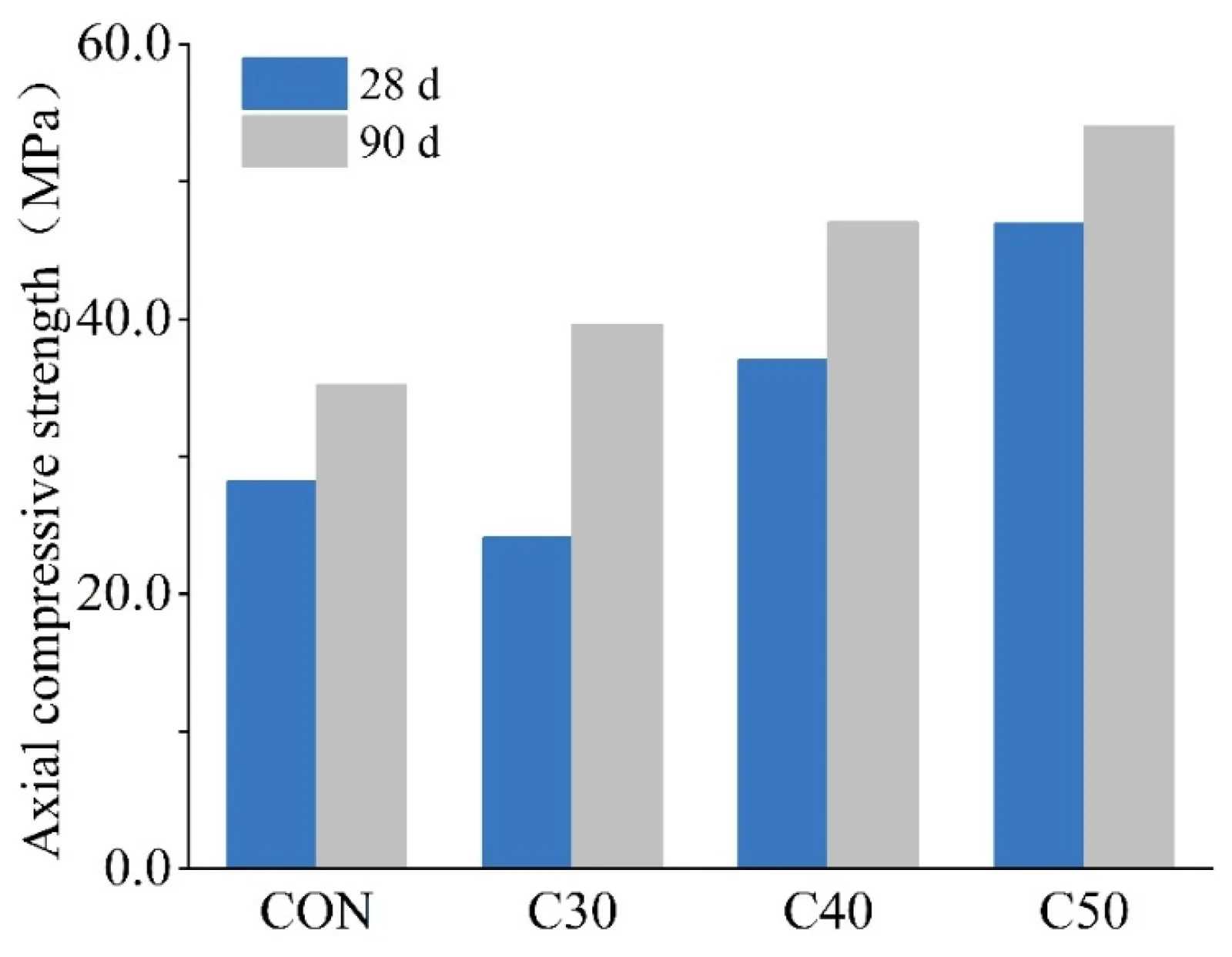
Axial compressive strength of concrete at 28 d and 90 d.

Figure 7 presents the axial compressive strength of all concrete groups at 28 d and 90 d. The evolution of axial compressive strength with curing age followed trends similar to those of compressive strength and splitting tensile strength, with values rising as curing time extended. In addition, axial compressive strength increased with the strength grade of CWS concrete at all tested ages, which was consistent with the variation law of conventional river sand concrete. At 28 d, the C30 group of CWS concrete had lower axial compressive strength than the control group of river sand concrete under the same mix proportions, with corresponding values of 24.09 MPa and 28.17 MPa. This trend reversed at 90 d, where the axial compressive strength of the CON group decreased below that of the C30 group, recording 35.18 MPa and 39.58 MPa respectively. From 28 d to 90 d, hydration decelerated in conventional river sand concrete, whereas the internal curing effect of CWS kept promoting cement hydration and thus sustained strength development.

Furthermore, axial compressive strength showed a positive correlation with compressive strength at the same curing age across all groups. In all cases, axial compressive strength values were lower than compressive strength. Owing to the greater length of axial compression test specimens, the hoop effect was significantly diminished in the middle portion. This region approached a true axial compression condition, thereby resulting in a lower axial compressive strength.

**Figure 8:**
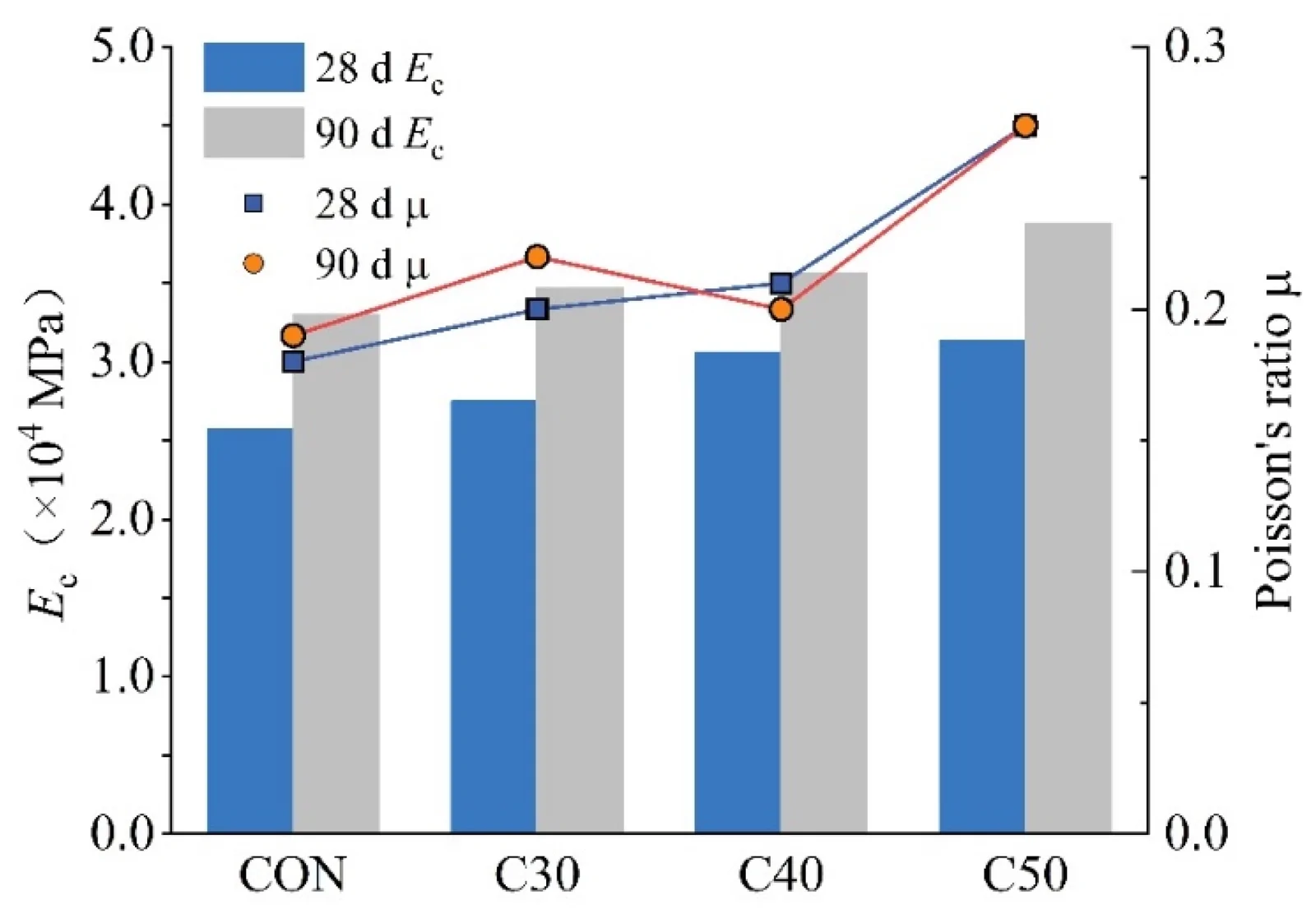
Static elastic modulus and Poisson’s ratio of different concrete mixtures.

According to the Poisson’s ratio data in Figure 8, no remarkable age-dependent variation was observed for all test groups. At the same curing age, Poisson’s ratio increased moderately with concrete strength. Under identical mix proportions, the C30 CWS concrete had a Poisson’s ratio close to that of the CON river sand concrete. The typical Poisson’s ratio of conventional concrete is 0.15–0.25 [37], while the values of CWS concrete in this study fell within 0.20–0.27, showing a slight increase. The discrepancy could be attributed to the different physical characteristics of the fine aggregates used.

**Figure 9:**
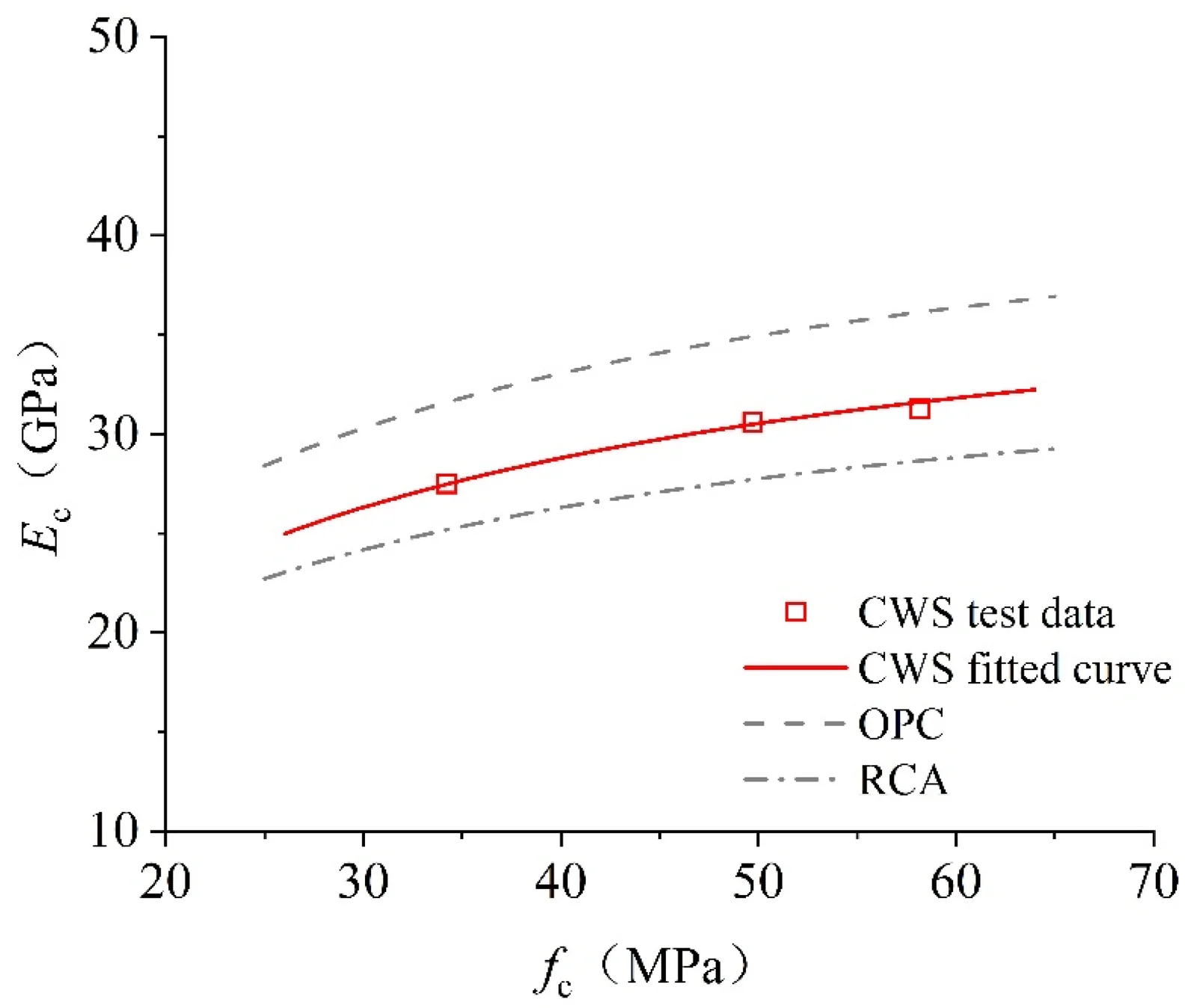
Relationship between static elastic modulus and compressive strength.

As shown in Figure 8, the static elastic modulus of each concrete group grew with curing age, which agreed with the variation law of conventional concrete. For CWS concrete, the static elastic modulus also rose as the strength grade increased. At 28 d and 90 d, the C30 group of CWS concrete exhibited a slightly higher static elastic modulus than the normal river sand concrete of the CON group with identical mix proportions. As listed in Table 3, numerous studies have established correlations between static elastic modulus (*E*_c_) and compressive strength (*f*_c_) for conventional concrete [38,39,40,41,42,43]. However, due to differences in aggregate characteristics, existing formulas for conventional concrete were not applicable for CWS concrete and thus required revision. Referring to the formula for ordinary Portland cement (OPC) concrete specified in the Chinese Standard GB/T 50010-2010 [38] and the equations for recycled concrete aggregate (RCA) concrete proposed by Xiao et al. [44], regression analysis was conducted on the *E*_c_-*f*_c_ relationship of CWS concrete based on the experimental data. The derived Equation (9) had a correlation coefficient R^2^ of 0.99, reflecting a satisfactory fitting effect. Detailed results are presented in Figure 9.

(9) $$E_{c}=\frac{10^{5}}{2.48+\frac{39.54}{f_{c}}}$$

In Figure 9, the red scatter points denote the measured data of CWS concrete, and the solid red line corresponds to the fitted regression function, namely Equation (9). The gray dashed line and gray dotted line represent the regression relationships between static elastic modulus (*E*_c_) and compressive strength (*f*_c_) for OPC concrete and RCA concrete, respectively [38,44]. Comparative analysis indicated that the static elastic modulus of CWS concrete was lower than that of OPC concrete but higher than that of RCA concrete. Since the static elastic modulus of concrete is predominantly governed by aggregate characteristics, including surface morphology, particle shape, size, and stiffness, it can be inferred that the overall stiffness of CWS aggregates is lower than that of natural river sand aggregates but superior to that of recycled concrete aggregates.

### 4.2. Durability Properties

#### 4.2.1. Sorptivity

**Figure 10:**
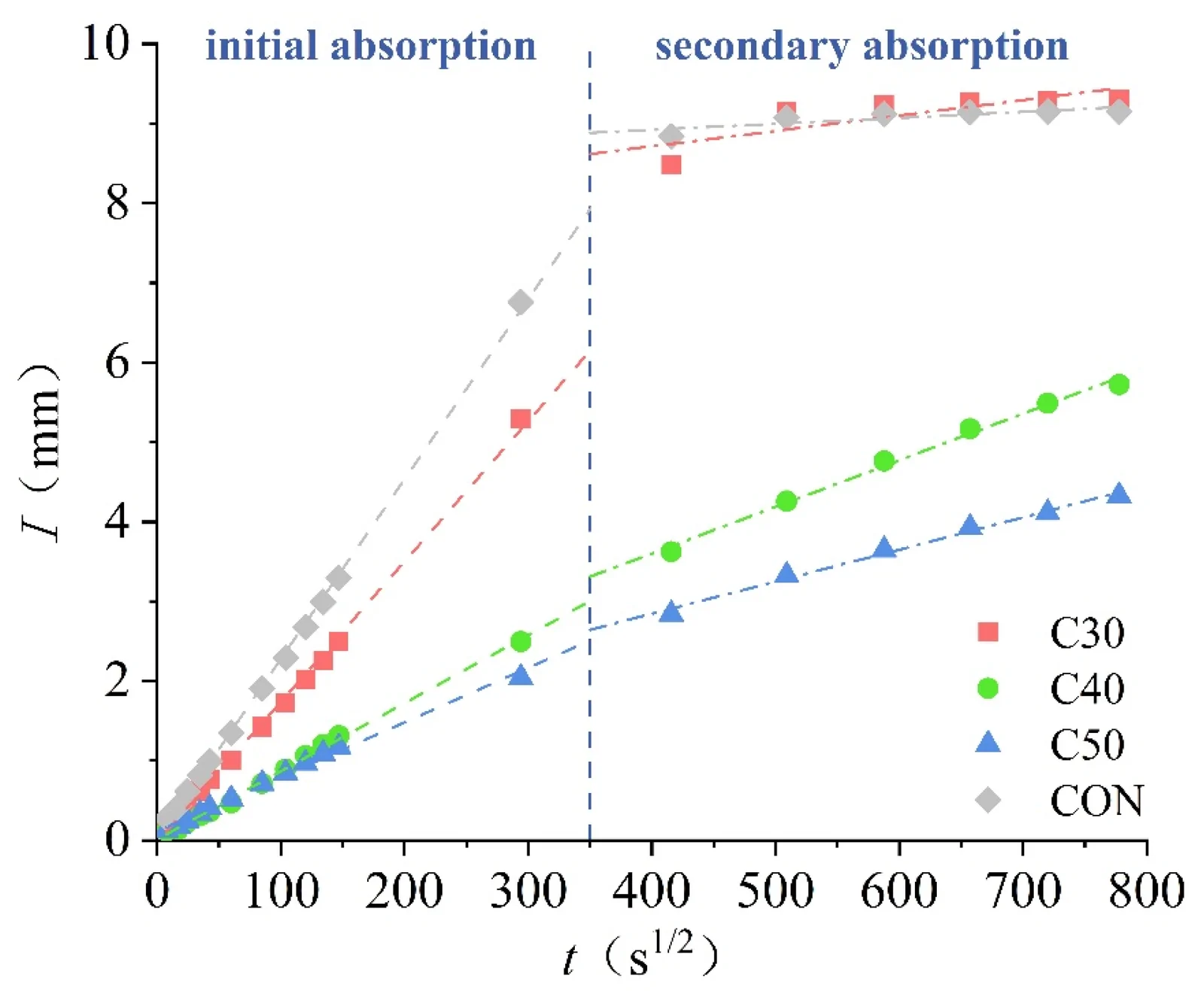
Sorptivity of concrete mixtures at the testing age of 28 d.

**Figure 11:**
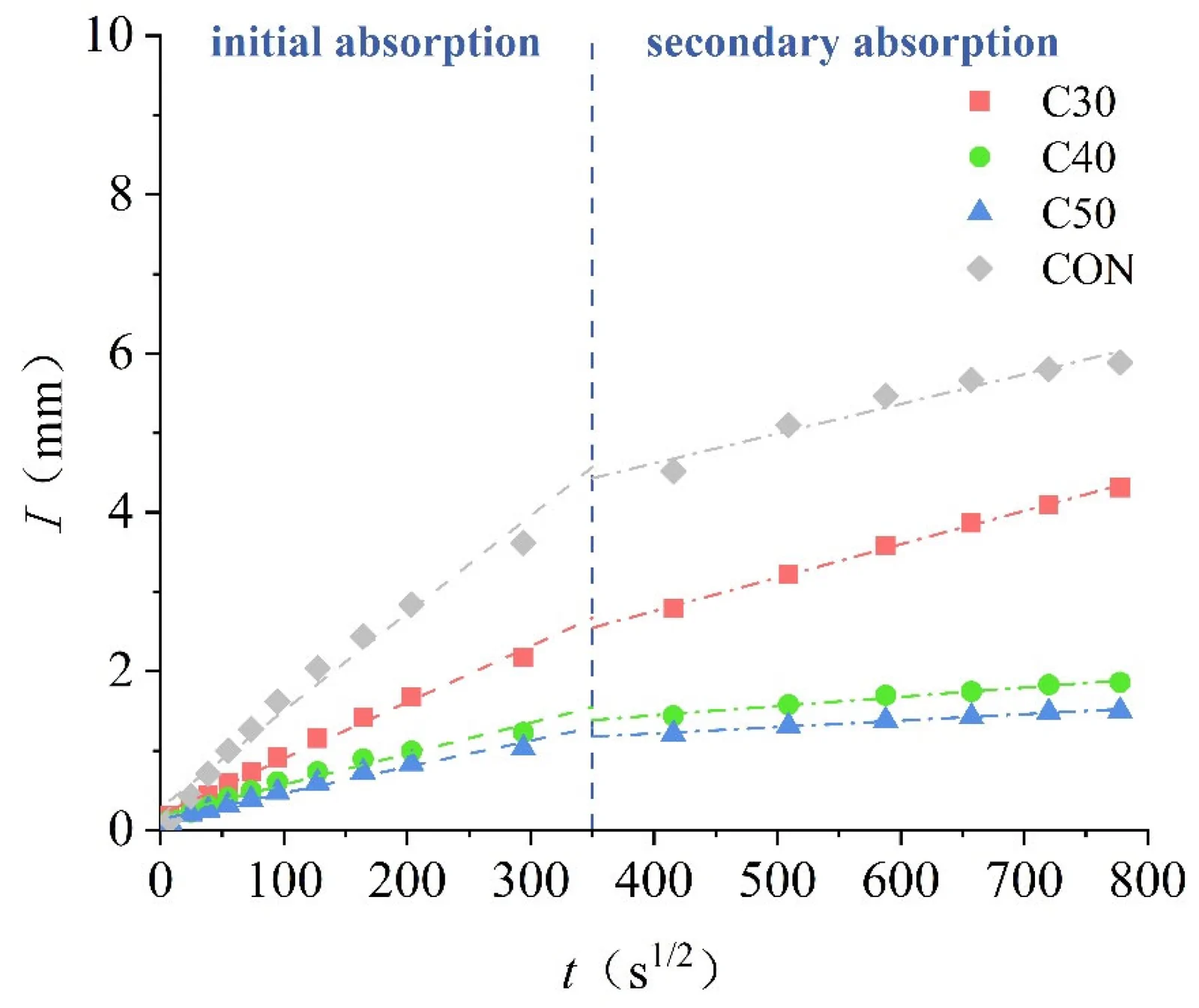
Sorptivity of concrete mixtures at the testing age of 90 d.

In real marine environments, harmful ions including Cl^−^ and SO_4_^2−^ penetrate into concrete structures with water acting as the transport medium. Accordingly, water absorption capacity serves as a key indicator for evaluating concrete durability [45]. Following the test procedure described before, experimental results were plotted with the square root of absorption time on the *x*-axis and water absorption on the *y*-axis. Figure 10 and Figure 11 present the sorptivity of all concrete mixtures at 28 d and 90 d, respectively.

As shown in Figure 10 and Figure 11, all concrete mixtures presented an obvious two-stage water absorption characteristic at both 28 d and 90 d. Water absorption rose rapidly at the initial stage and then slowed down gradually at the later stage. In this study, 1 day was set as the critical time point. The period within 1 d was defined as the initial absorption stage, while the duration from 1 d to 7 d was regarded as the secondary stage. Regression analysis was conducted on the data collected from these two stages. The slope of the fitted curve was defined as the water absorption coefficient (S), where S1 and S2 represented the initial and secondary water absorption coefficients, respectively. Detailed fitting results are listed in Table 4. The coefficient of determination R^2^ exceeded 0.95 for most groups, suggesting a satisfactory fitting quality.

As shown in Figure 10 and Figure 11, cumulative water absorption increased over time for all specimens at both 28 d and 90 d. Driven by capillary suction, water was absorbed rapidly at the initial stage. The absorption rate decreased remarkably in the secondary stage, which could be explained by two factors. First, water transport was hindered when encountering gel pores and other impermeable pores finer than capillary pores. Second, continuous water migration inside the specimens increased friction between water and capillary walls, further slowing down the absorption process. From 28 d to 90 d, the cumulative water absorption of each group declined evidently at identical testing time points, which complied with the variation trend of conventional river sand concrete. As reported in previous literature, surface capillary pores quickly reached saturation in the initial absorption stage, whereas the secondary stage was dominated by slow water adsorption in isolated pores under long-term capillary effects [46].

Furthermore, as shown in Table 4, the primary water absorption coefficient of CWS concrete at 28 d decreased with increasing strength grade. This trend agreed with that of conventional concrete, revealing that higher-strength CWS concrete possessed a denser internal microstructure. For mixtures with identical mix proportions, the initial and secondary water absorption coefficients showed no obvious difference between C30 group of CWS concrete and the conventional river sand concrete of the CON group, which corresponded well to their compressive strength performance at 28 d. This reflected similar density and porosity between the two groups. Nevertheless, the secondary water absorption coefficients of C40 and C50 groups were remarkably higher than those of the lower-strength C30 and CON groups. This phenomenon was attributed to the higher cement dosage adopted in C40 and C50 mixtures, which generated more gel pores. Continuous hydration produced additional hydration products and further refined the pore structure. By 90 d, the initial water absorption coefficients of all groups decreased substantially relative to the values at 28 d. Specifically, the coefficient of the CON group dropped by 45.71%, while the reduction rates reached 59.84%, 54.50% and 51.82% for the C30, C40 and C50 groups, respectively. Meanwhile, the secondary water absorption coefficients of C40 and C50 also decreased evidently and became distinctly lower than those of C30 and CON groups under the same mix proportions. The results demonstrated that a higher dosage of cementitious materials effectively filled internal pores in CWS concrete. In addition, CWS aggregates exerted an internal curing effect, which highlights the importance of proper curing for CWS concrete. At 90 d, the C30 group exhibited lower cumulative water absorption than the CON group, proving that CWS concrete had better durability compared with conventional river sand concrete under identical mix proportions.

#### 4.2.2. Chloride Ion Penetration

**Figure 12:**
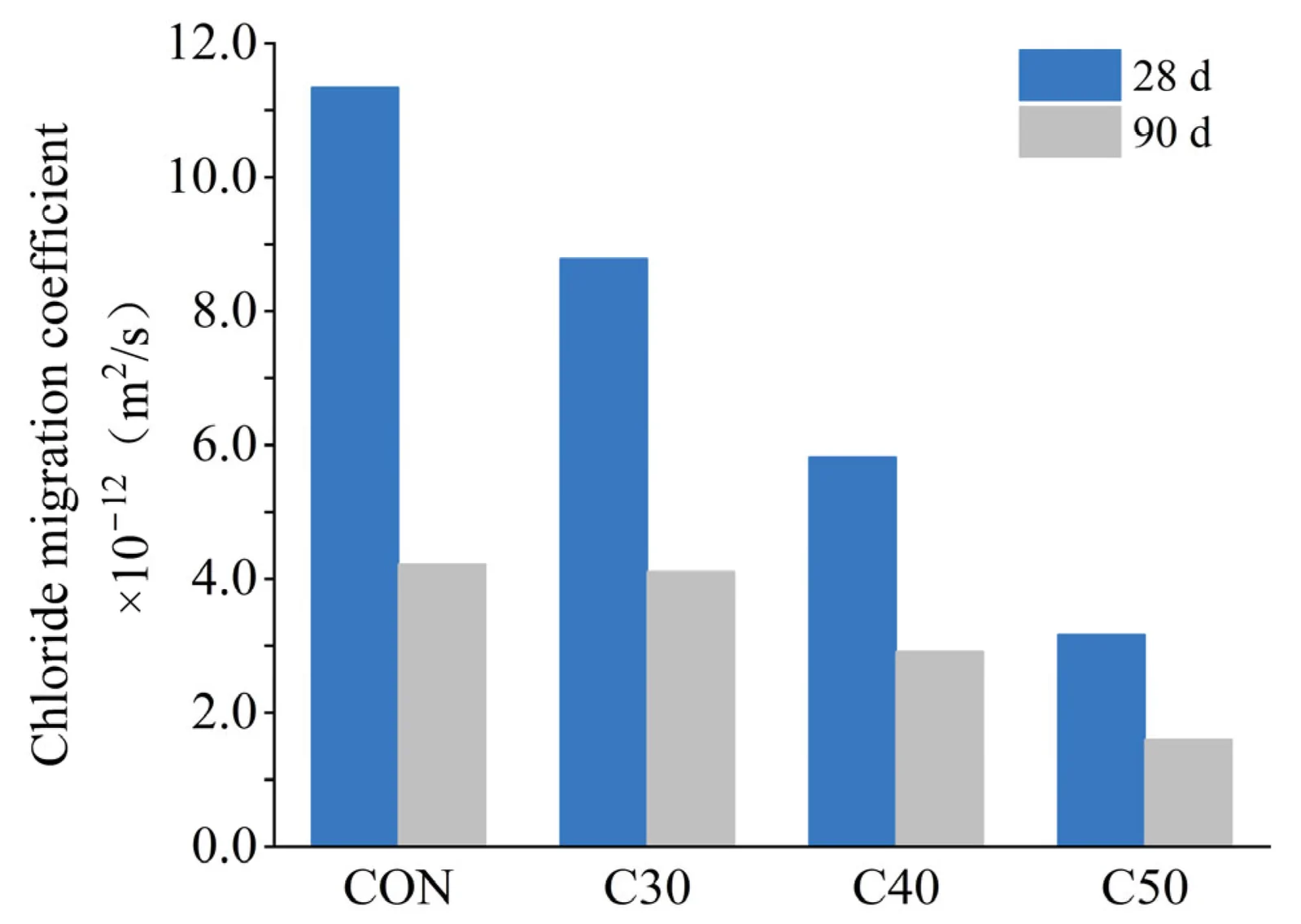
Chloride migration coefficient of concrete mixtures at 28 d and 90 d.

According to the resistance of concrete to chloride ion penetration, the durability of concrete can be described by the chloride migration coefficient [47]. The computed chloride migration coefficient of different specimens at 28 d and 90 d is presented in Figure 12. At 28 d, the C30 group of CWS concrete exhibited a notably lower chloride migration coefficient than the CON group of normal river sand concrete under identical mix proportions. The porous structure of CWS aggregates enabled effective physical adsorption of harmful ions, which was favorable for reinforced concrete structures serving in harsh marine environments. As hydration proceeded, hydration products gradually filled internal pores and microcracks, densifying the concrete matrix and enhancing its resistance to chloride ingress. Accordingly, all groups of specimens achieved a distinct reduction in the chloride migration coefficient from 28 d to 90 d. The chloride migration coefficient of the CON group dropped by 62.8% at 90 d compared to 28 d, while the reductions for the C30, C40 and C50 groups reached 53.2%, 50.0% and 49.3%, respectively. At 90 d, the chloride migration coefficients of the CON and C30 groups were 4.22 × 10^−12^ m^2^/s and 4.11 × 10^−12^ m^2^/s, showing no significant difference. For CWS concrete, the chloride migration coefficient declined with curing age, indicating that longer curing time contributed to better resistance against chloride penetration. In addition, at both 28 d and 90 d, the chloride migration coefficient of CWS concrete declined with the increase in strength grade, which was consistent with the behaviour of normal river sand concrete. Based on the foregoing mechanical test results, lower strength corresponded to a more porous internal structure, which provided more pathways for chloride transport and thus degraded chloride penetration resistance. It can therefore be concluded that the C50 group of CWS concrete possessed lower porosity than the C40 and C30 groups.

#### 4.2.3. Drying Shrinkage

**Figure 13:**
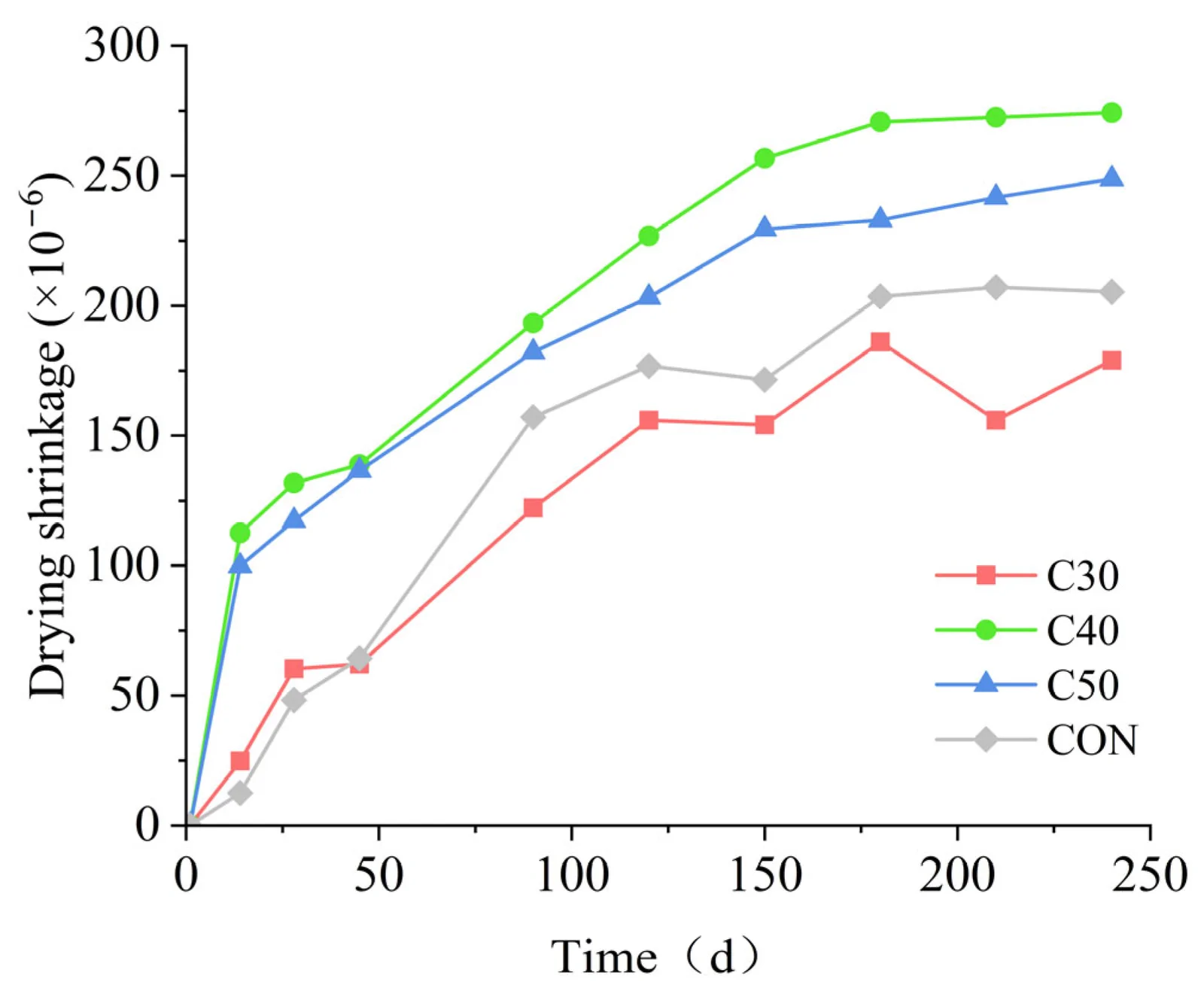
Drying shrinkage of concrete mixtures measured for 240 d.

Drying shrinkage property serves as a critical index for evaluating the durability of cement-based materials, which can induce volume shrinkage and cracking in concrete [48]. The drying shrinkage development in all concrete mixtures over time is shown in Figure 13. One important source of the drying shrinkage is the loss of water held in the capillary pores of concrete mixtures including both cement pastes and the CWS aggregates [49]. Thus, excluding minor measurement errors at individual testing ages, the value of drying shrinkage for all concrete mixtures increased with curing time going on. The evolution of drying shrinkage curves can be roughly divided into two stages during 240 days. The first 180 days are the first stage in which the growth rate of length change was rather high, while the growth rate slowed down in the following second stage. This difference was mainly attributed to the various cement hydration rates and water evaporation processes in these two stages [50].

For mixtures with identical mix proportions, the C30 group of CWS concrete and the CON group of normal river sand concrete showed similar drying shrinkage rates within 45 days. After 90 days, the C30 group achieved a lower shrinkage rate, demonstrating that the drying shrinkage performance of CWS concrete was better than that of normal river sand concrete, and its durability was relatively better. This finding was consistent with the conclusions of the chloride ion penetration test. The rough surface morphology, high internal porosity and water absorption of CWS aggregates did not exert an adverse effect on drying shrinkage performance. Unlike normal river sand, moisture existed not only in the cement paste but also within the pores of CWS aggregates. As hydration products gradually filled these pores, the evaporation of internal moisture was effectively retarded. In addition, for CWS concrete of different strength grades, the drying shrinkage rate rose with strength at all testing ages, following the same trend as normal river sand concrete. This is primarily because higher-strength mixtures such as C50 contained more cementitious materials. It is widely acknowledged that concrete with a higher cementitious content tends to develop greater drying shrinkage [51].

### 4.3. Microscopic Mechanism

#### 4.3.1. Pore Size Distribution

**Figure 14:**
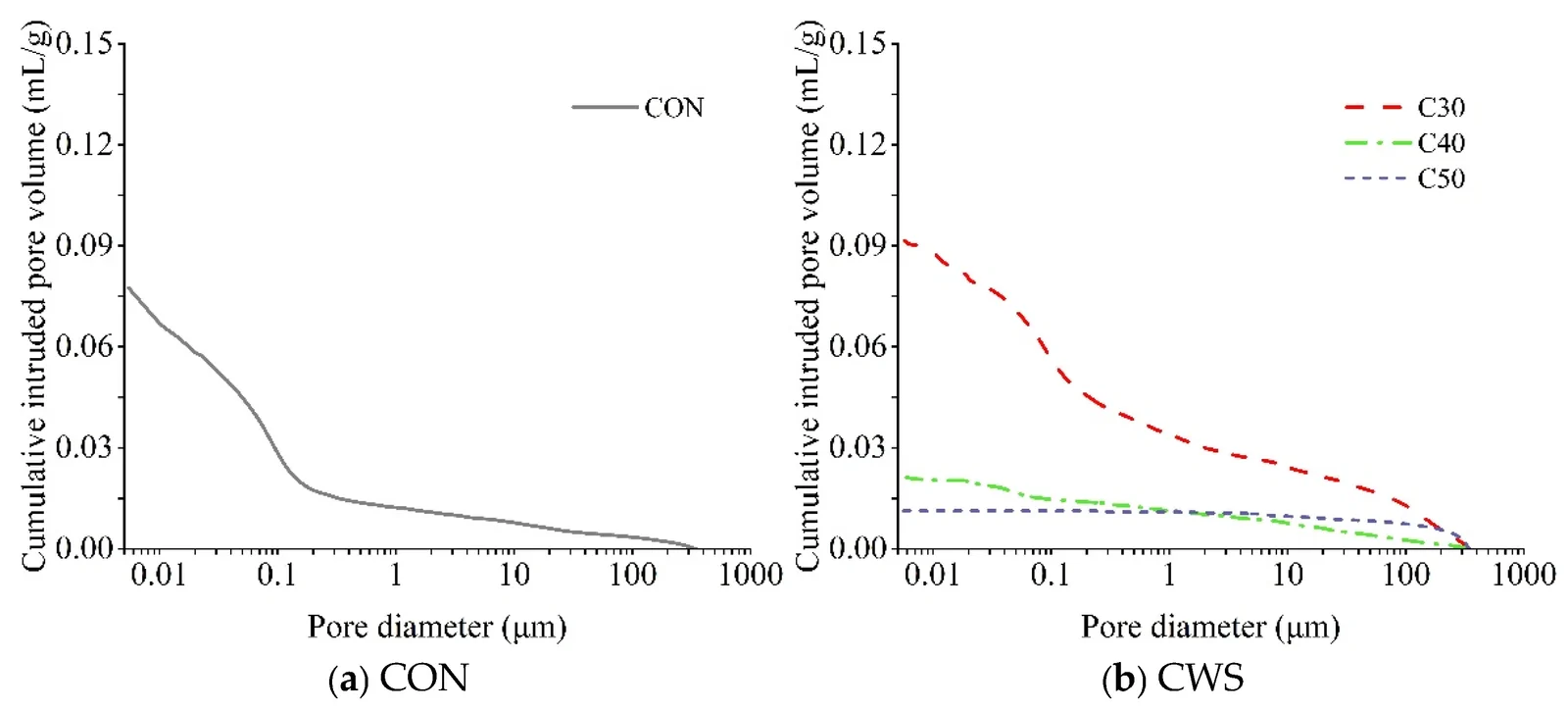
Cumulative intrusion curve of the concrete at 28 d.

The pore distribution of concrete has a significant impact on its mechanical and durability properties [52]. In this section, the pore structure of CON and CWS concrete with various strength grades at the same curing age is discussed based on the MIP results. Figure 14 shows the cumulative intrusion curves as a function of pore diameter for all tested groups at 28 d. Comparing Figure 14a,b, the cumulative intrusion curves of the CON and C30 groups exhibited similar trends, with no prominent distinction in total porosity. At 28 d, CWS concrete exhibited marginally higher porosity than normal river sand concrete prepared with the same mix proportions, which originated from the inherent physical discrepancy of fine aggregates. As reported in existing literature [53], reduced porosity corresponds to denser matrix and superior mechanical and durability performance. This accounts for the slightly lower compressive strength for the C30 group of CWS concrete compared with the CON group at 28 d. Meanwhile, the relatively higher pore content enables enhanced adsorption of detrimental ions, leading to a marginally smaller chloride migration coefficient for CWS concrete. As illustrated in Figure 14b, porosity declined markedly with the rising strength grade of CWS concrete at the same curing age. No obvious porosity difference was detected between the C40 and C50 groups of CWS mixtures, implying comparable matrix density. Accordingly, C40 and C50 specimens presented close values in compressive strength and water absorption coefficient at 28 d. This result was consistent with the other mechanical and durability properties analyzed before.

**Figure 15:**
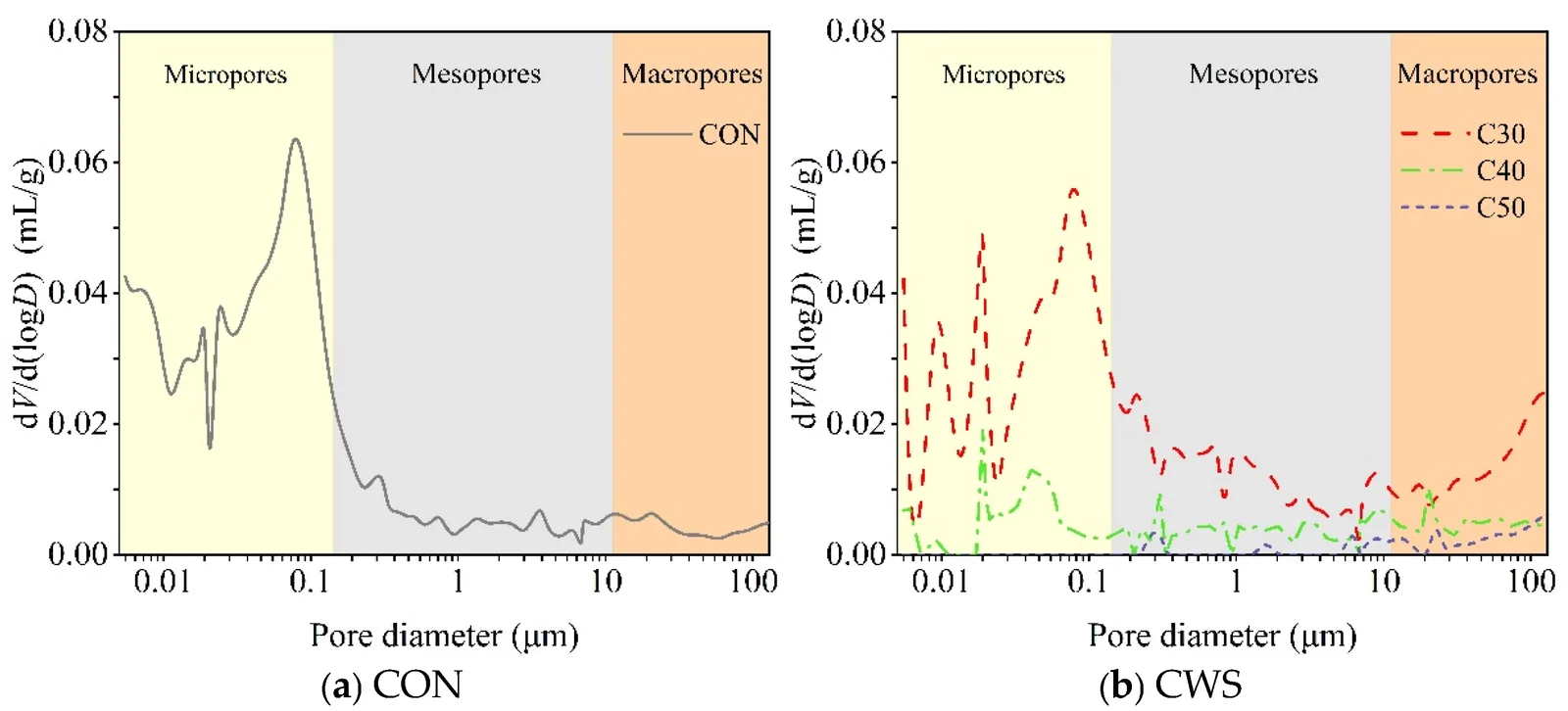
Pore size distribution curve of the concrete at 28 d.

To further clarify the pore structure of all concrete mixtures at 28 d, the pore size distribution curves representing logarithmic differential intruded volume versus pore diameter are presented in Figure 15. In accordance with the classification proposed in Reference [54], concrete pores are categorized into three grades by pore diameter. Micropores correspond to diameters ranging from 0.001 to 0.1 mm, mesopores fall within 0.1–10 mm, and macropores are defined as pores exceeding 10 mm. A comparison of curves in Figure 15a,b for the CON and C30 groups reveals nearly identical micropore distribution for CWS concrete and normal river sand concrete with the same mix proportions. Distinctions primarily emerged within the mesopore and macropore ranges, where CWS concrete contained a higher fraction of meso- and macropores owing to inherent discrepancies in fine aggregate characteristics. CWS fine aggregates inherently possess abundant meso- and macropores, leading to greater contents of such pores in corresponding concrete compared with river sand concrete of identical mix proportions. Moreover, as illustrated in Figure 15b, the pore size distribution curve of CWS concrete gradually shifted downward with rising strength grade at a fixed curing age. This trend demonstrates that, consistent with conventional concrete, higher strength grades corresponded to denser matrix and lower total porosity for CWS concrete [55].

#### 4.3.2. Mineralogy and Microstructure Analysis

**Figure 16:**
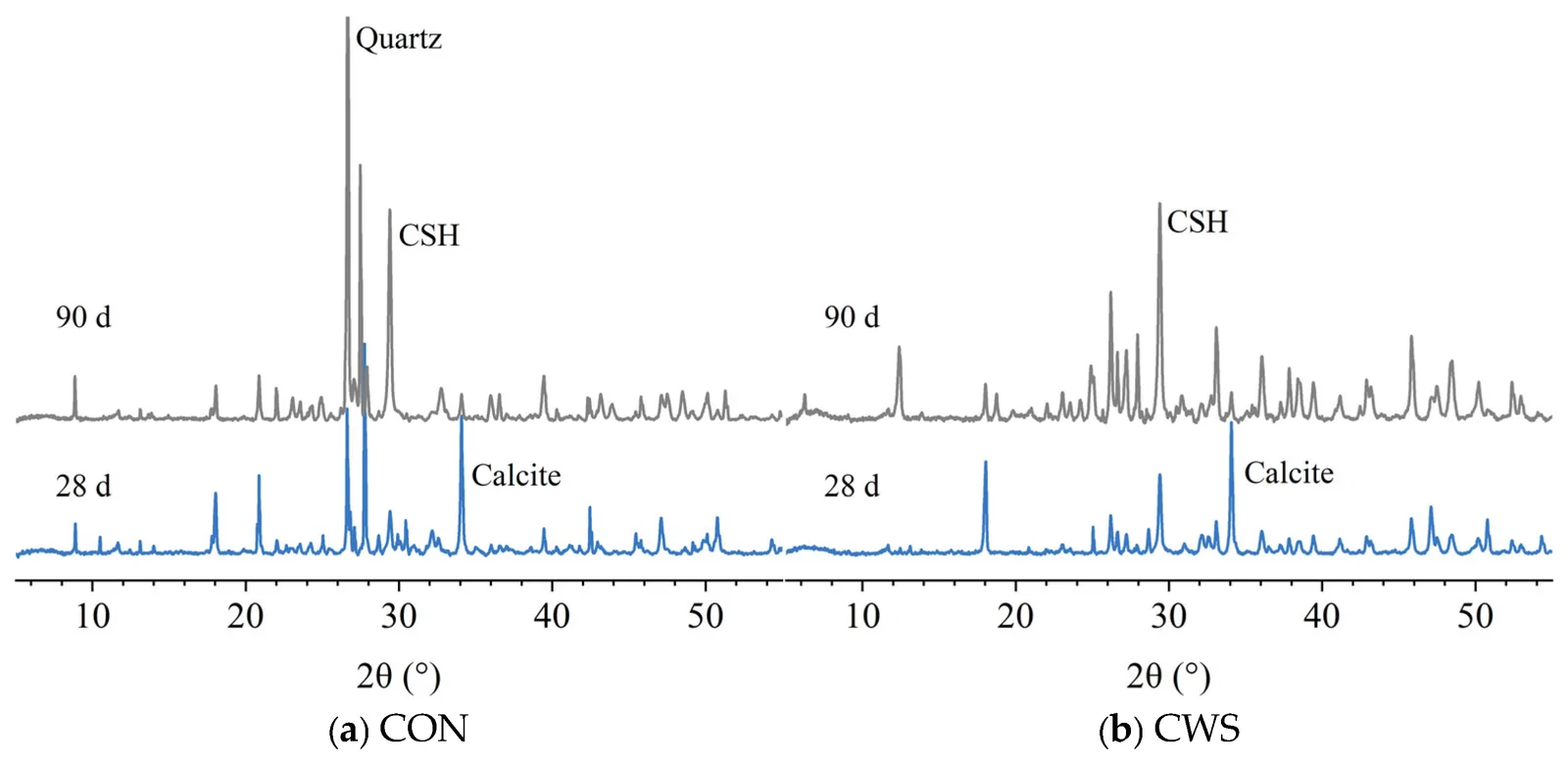
XRD pattern of the concrete at 28 d and 90 d.

The samples were characterized using XRD to study the mineralogical changes in concrete with different fine aggregates. The XRD results of CON and CWS concrete at 28 d and 90 d are shown in Figure 16. Notably, the CWS concrete used here is the C30 group of CWS concrete with the same mix proportion as the CON group of normal river sand concrete. Comparing Figure 16a,b, diffraction peaks corresponding to quartz and calcium carbonate were detected in CON concrete. The quartz peak stemmed from natural river sand, whereas calcium carbonate originated from limestone filler. By contrast, only prominent calcium carbonate diffraction peaks appeared in CWS concrete. As reported in previous studies [56], both CON and CWS concrete exhibited weak calcium silicate hydrate (C-S-H) diffraction peaks at 28 d. After 90 days of curing, the C-S-H peak intensity rose considerably for both mixtures, demonstrating ongoing cement hydration accompanied by continuous generation of hydration products and progressive improvement in hydration degree. Yang et al. [57] confirmed that curing age significantly affects hydration product formation, and longer curing contributes to more abundant hydration phases. These newly formed products refine the internal pore structure, densify the matrix, and enhance the mechanical and durability performance of concrete. Accordingly, prolonged curing led to increased compressive and splitting tensile strengths for all CON and CWS mixtures, alongside reductions in water absorption coefficient and chloride migration coefficient.

**Figure 17:**
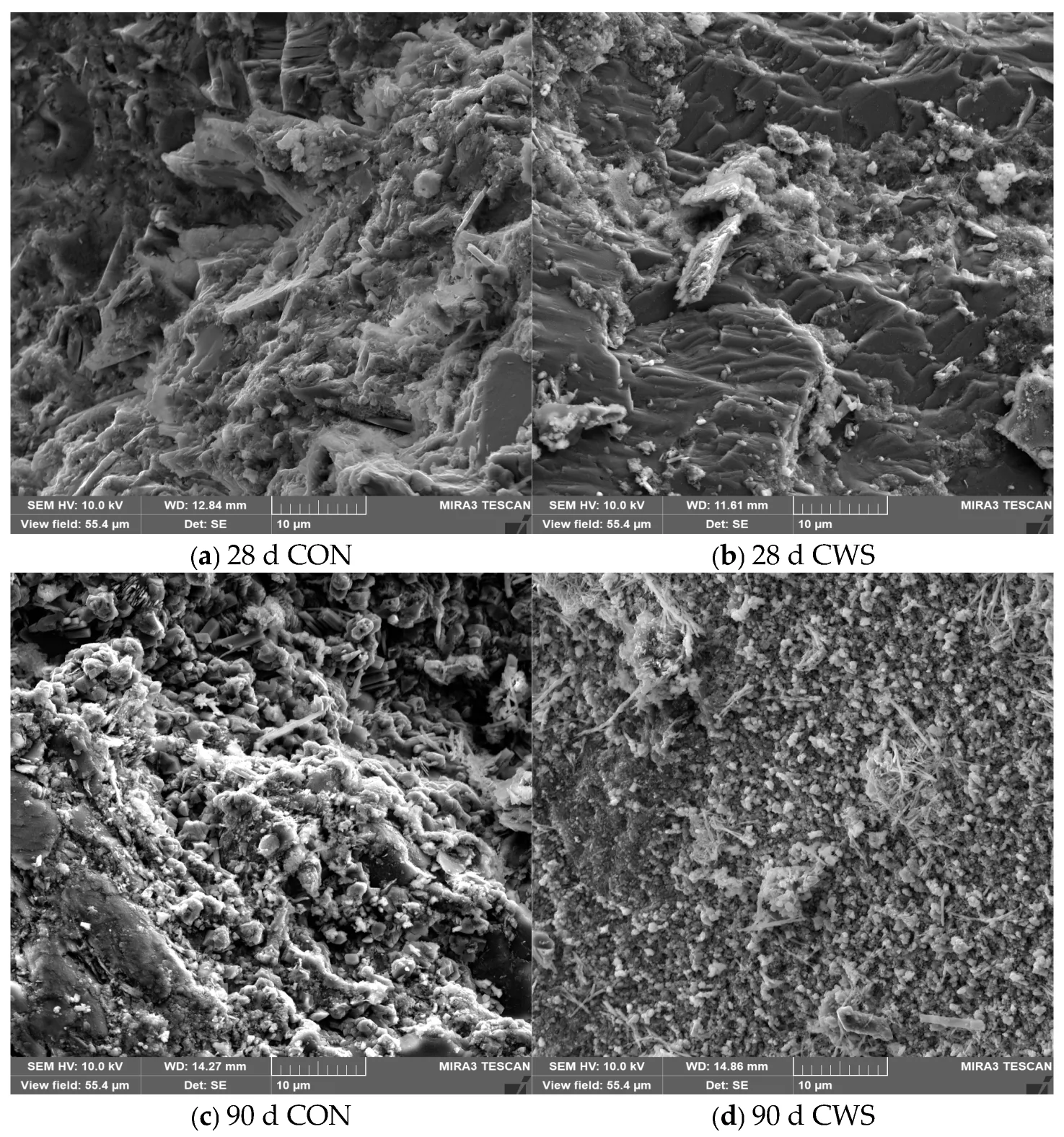
SEM images of the concrete at 28 d and 90 d. (5000×).

The microstructure of CWS concrete and control concrete with the same mix proportion was observed by SEM at 28 d and 90 d. SEM images of CON and CWS concrete at magnifications of 5000× are shown in Figure 17a–d, separately. From Figure 17a,b, floc-like C-S-H gels and tabular portlandite (CH) crystals coated the aggregate surfaces in both CON and CWS concrete specimens at 28 d. The quantity of hydration products increased substantially at 90 d for both concrete mixtures, in good accordance with the foregoing pore structure and XRD analysis results.

## 5. Conclusions

This study aims to investigate the possibility of fully utilizing crushed waste shells (CWS) as all fine aggregate in the preparation of eco-friendly concrete with different strength grades and satisfactory workability. In this study, three groups of eco-friendly CWS concrete (C30, C40 and C50) were made instead of direct sand replacement based on normal river sand concrete. Meanwhile, a control mixture incorporating full natural river sand as fine aggregates was occupied for comparation, which had the same mixing proportion as the C30 group of CWS concrete. Besides mechanical strength and durability-related transport properties, pore structure distribution, mineral composition and microstructure of CWS concrete were investigated and compared to the control concrete. Key conclusions derived from the whole experimental results are summarized as follows.

(1) Experimental results demonstrated that CWS can fully replace river sand to fabricate concrete satisfying the designed strengths of C30, C40 and C50, but it is not a simple one-to-one replacement according to the normal river sand concrete mix proportion. Considering the high porosity and water absorption of CWS aggregates, pre-wetting pretreatment of aggregates and an increased sand ratio are recommended for practical engineering according to the optimized mix proportions developed in this work.

(2) For mechanical performance, CWS concrete exhibited an identical strength-gain trend to normal river sand concrete, with strength rising continuously as curing proceeded. The Poisson’s ratio of C30-C50 groups of CWS concrete ranged from 0.20 to 0.27. In contrast to Poisson’s ratio, the static elastic modulus of CWS concrete was more sensitive to variations in curing age.

(3) In terms of durability, the CWS concrete proposed in this study exhibited better durability performance than normal river sand concrete with identical mix proportions. Especially at early curing ages, the chloride ion migration coefficient and water absorption coefficient of CWS concrete were lower than those of the proportion-matched river sand concrete. This advantage benefits reinforced concrete structures serving in harsh marine environments and effectively delays the arrival of detrimental ions at the surface of steel reinforcement. In addition, consistent with normal river sand concrete, higher-strength CWS concrete featured a denser matrix, yet these mixtures are susceptible to high shrinkage. In practical applications, attention should be paid to the curing regimes or binder system modification of high-strength CWS concrete to limit early-age drying shrinkage.

(4) Compared with conventional river sand, CWS fine aggregate contained abundant intrinsic pores that act as built-in water reservoirs. The absorbed water was gradually released throughout the cement hydration process to deliver an internal curing effect. Such internal curing effect accelerated ongoing hydration, refined the pore structure of CWS concrete, and improved its mechanical and durability properties compared with normal river sand concrete of identical mix proportions. This independent internal curing mechanism was verified only for the C30 series, while the higher-strength grades arose from a coupled effect of multiple variables.

It should be noted that soluble salt analysis was not conducted on the raw crushed waste shells in this work. Therefore, all grades of CWS concrete (C30–C50) reported herein are recommended for non-structural applications only. In addition, the present study primarily investigates the feasibility of producing CWS concrete with strength grades of C30–C50 using crushed waste shells as a 100% fine-aggregate replacement. Further low-carbon optimization of the cementitious system is worthy of consideration in future investigations. For instance, the incorporation of supplementary cementitious materials to partially replace ordinary Portland cement can effectively reduce carbon emissions and improve the economic performance of CWS concrete, thereby facilitating the practical engineering application of such eco-friendly concrete.

## Figures and Tables

**Table 1:** Chemical compositions of OPC (P.O. 42.5).

Chemical Compositions (%)							
CaO	SiO_2_	Al_2_O_3_	Fe_2_O_3_	SO_3_	Na_2_O	K_2_O	MgO
60.16	21.35	4.94	2.71	1.96	1.00	0.48	0.46

**Table 2:** Concrete mix proportions.

Sample		Material Consumption/($kg\cdot m^{-3}$)					Net w/c	Total w/c	Slump mm
	Cement	CWS	River Sand	Coarse Aggregates	SP	Water			
C30	550	786	/	786	0	279	0.45	0.507	51
C40	700	659	/	805	3.5	236	0.30	0.338	84
C50	850	572	/	700	8.5	278	0.30	0.327	73
CON	550	/	786	786	0	279	0.45	0.507	75

**Table 3:** Static elastic modulus formulas for ordinary concrete.

No.	Formula	Source	Reference
1	$E_{c}=\frac{10^{5}}{2.2+\frac{33}{f_{c}}}$	China	[38]
2	$E_{c}=\frac{10^{5}}{1.7+\frac{36}{f_{c}}}$	USSR	[39]
3	$E_{c}=4127\sqrt{f_{c}}$	USA	[40]
4	$E_{c}=\sqrt[{3}]{0.1\;f_{c}+0.8}\times 2.15\times 10^{4}$	EU	[41]
5	$E_{c}=370\;f_{c}+13,100$	Dhir	[42]
6	$E_{c}=378\;f_{c}+8242$	Mellmann	[43]

**Table 4:** Fitting results of water absorption curves.

Sample	Testing Age	*S*_1_/ $\mathrm{mm}\cdot s^{-0.5}$	R^2^	*S*_2_/ $\mathrm{mm}\cdot s^{-0.5}$	R^2^
C30	28 d	0.01763	0.997	0.00193	0.667
	90 d	0.00708	0.992	0.00421	0.998
C40	28 d	0.00855	0.998	0.00585	0.993
	90 d	0.00389	0.972	0.00117	0.981
C50	28 d	0.00685	0.994	0.00405	0.992
	90 d	0.00330	0.980	0.00081	0.985
CON	28 d	0.02260	0.999	0.00075	0.690
	90 d	0.01227	0.976	0.00374	0.942

## Data Availability

The original contributions presented in this study are included in the article. Further inquiries can be directed to the corresponding author.
